# Neutrophil-mediated and low density lipoprotein receptor-mediated dual-targeting nanoformulation enhances brain accumulation of scutellarin and exerts neuroprotective effects against ischemic stroke†

**DOI:** 10.1039/c8ra06688d

**Published:** 2019-01-10

**Authors:** Yanxin Dang, Chiying An, Yutao Li, Dandan Han, Xin Liu, Fengming Zhang, Yuan Xu, Haijing Zhong, Mewand Khan Karim Khan, Fengjuan Zou, Xiaojun Sun

**Affiliations:** Department of Pharmaceutical Engineering, School of Chemical and Environmental Engineering, Key Laboratory of Green Chemical Engineering, Harbin University of Science and Technology Harbin China xinliu98@126.com xin.liu@yale.edu sunxiaojun361@163.com; Heilongjiang Province Rehabilitation Hospital Harbin China; The First Affliated Hospital of Harbin Medical University Harbin China; Department of Pharmacology, School of Medicine, Yale University New Haven Connecticut USA

## Abstract

Delivery of poorly permeable drugs across the blood-brain barrier (BBB) is a great challenge in the treatment of ischemic stroke. In order to construct a suitable delivery system for this purpose, we developed a dual-targeting nanoformulation to transfer therapeutic agents targeting the inflammatory sites of the ischemic brain. The matrix of this system is a hydroxyl-terminated polyamidoamine dendrimer with excellent biodegradability. The surface of the matrix is functionalized with two targeting peptides: Angiopep-2 is a low density lipoprotein receptor-mediated peptide with high BBB transcytosis capacity with ligands expressed on brain endothelial cells; N-acetylated proline-glycine-proline (PGP) has high affinity to CXCR2 expressed on infiltrating neutrophils. This system proved to be a high-loading formulation for the neuroprotective compound, scutellarin (STA), and significantly improved its therapeutic efficacy in a rodent model of ischemic stroke. The molecular mechanism underlying the therapeutic efficacy of this formulation is associated with significant down-regulation of the inflammatory cytokines, neutrophils infiltration and intracellular calcium overload and blockade of the inflammatory signaling pathway HMGB1/TLRs/MyD88/TRIF/NF-κB. Our results suggest that this dual-targeting delivery system is a promising drug delivery vehicle for ischemic stroke, and possibly other CNS diseases where neuroinflammation is involved.

## Abstract 2

Delivery of poorly permeable drugs across the blood-brain barrier (BBB) is a great challenge in the treatment of ischemic stroke.

## Graphical abstract

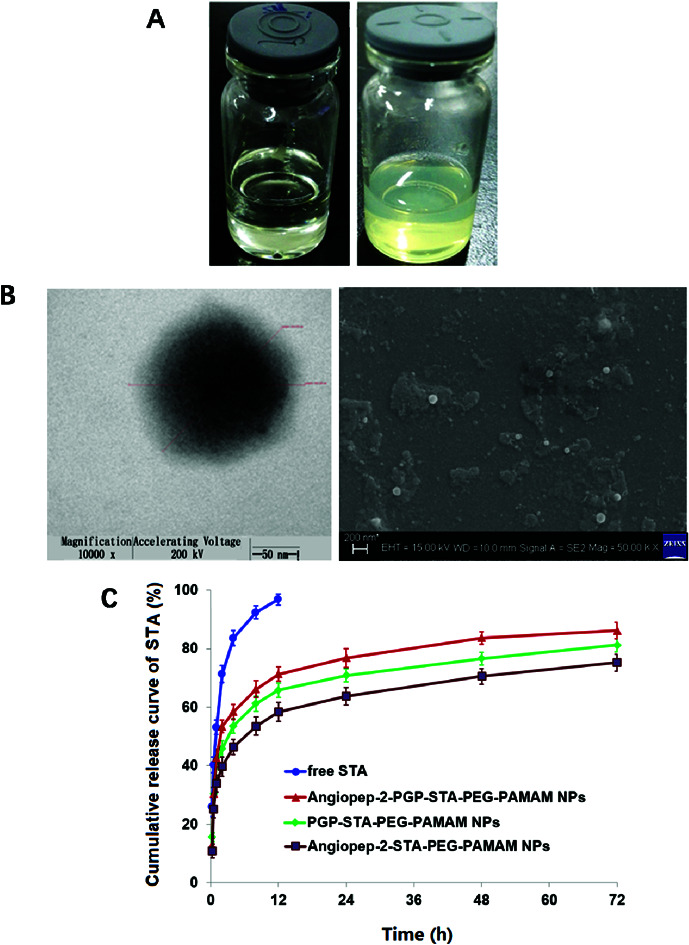

## 1. Introduction

Ischemic stroke is a leading cause of death and the most common cause of long-term disability in adults worldwide.^1^ However, the therapeutic effect of many drugs remain unsatisfactory for its clinical treatment, most often due to their low permeation across the BBB. Although there is a compromised endothelial barrier which facilitates molecular transport under some disease conditions such as Alzheimer's disease and ischemic stroke, the BBB is still present in the infiltrating margin of brain diseases.^2^ Our previous work demonstrated that the effective therapeutic strategy for ischemic stroke requires nanoparticulate drug delivery systems (nano-DDS) that can penetrate the blood–brain barrier (BBB), specifically targeting the ischemic area and protecting the cerebral tissue against ischemia-reperfusion injury.^3^

Scutellarin (STA), a typical flavonoid (4,5,6-trihydroxyflavone-7-glucuronide), is a Chinese medicine ingredient traditionally used for treatment of acute cerebral infarction and subsequent paralysis (Fig. S1†). Modern pharmacological studies have demonstrated the protective effects of STA on cerebral ischemia/reperfusion (I/R) injury because of its anti-oxidative actions and anti-apoptotic properties, as well as its ability to attenuate microglial inflammatory response and neuronal damage.^4^ However, the clinical outcomes of commercially available STA formulations are not as satisfactory as expected due to the low drug loading capacity, fast metabolic rate and short blood residence time of STA *in vivo*.^5^ Moreover, STA has low solubility in both water and lipids. Low water solubility prevents STA from intravenous injection, and low lipid solubility may account for its poor permeability across the BBB.^6^ Therefore, it is urgent to develop a high-loading active-targeting nanoformulation for STA that can improve its intravenous bioavailability, prolong its circulation half-life, and increase its permeability across the BBB.

Nanotechnology represents a promising approach for the delivery of therapeutic agents to the brain.^7–9^ Hydroxyl-terminated G5.0 polyamidoamine dendrimers (G5.0 PAMAM), a series of highly branched, spherical macromolecules with well-defined size (3–12 nm), are increasingly recognized as novel multifunctional nanocarriers for the delivery and controlled release of biologically active substances. This is attributable to their capability of surface functionalization by either covalent bonding or physical absorption, as well as high loading capacity of therapeutic agents in the hydrophobic core. Some dendrimer formulations have been shown to cross the impaired BBB *in vivo*.^10,11^

Receptor-mediated endocytosis is one of the most important mechanisms for brain-targeting drug delivery systems. Low density lipoprotein receptor-related protein1 (LRP1) plays an active role in mediating the transport of numerous ligands across the BBB.^12^ Angiopep-2, a 19-amino-acid peptide derived from the common peptidic sequence of the LRP1 protein ligands, exhibits much higher BBB transcytosis efficacy and parenchymal accumulation than transferrin, lactoferrin and avidin.^2^ Nanoparticles modified with Angiopep-2 has been proved possessing an excellent ability to cross the BBB.^13^

Neutrophil infiltration is one of the most prominent histological features after cerebral ischemia.^3^ It has been generally accepted that neutrophils are the first blood-borne cells found in the cerebral ischemic area,^14^ reaching peak numbers at 2–4 days after transient ischemia and declining thereafter.^15^ Several investigations indicated that the systemic immune response induced by ischemic injury is related to the increase of the circulating neutrophil number in stroke patients,^16^ which are then recruited to the brain.^17^ CXCR2, a classic chemokine receptor, is prominently expressed on phagocytic cells, especially neutrophils,^18^ which can serve as a specific inflammatory targeting receptor in ischemic stroke. Notably, neutrophils are the most abundant leukocytes in the human body (∼50%), which is sufficient as carriers for drug delivery in the entire pathogenesis process during cerebral ischemia. *N*-acetylated proline-glycine-proline (*N*-Ac-PGP) has been recognized to be a key chemokine involved in inflammation and plays an important role in neutrophil infiltration.^19^ Furthermore, PGP exhibits high affinity and specificity to CXCR2 receptor and low risk of immunogenicity.^20^ Therefore, PGP peptide may be an ideal neutrophil anchoring peptide to achieve precise targeting to the inflammatory sites for treatment of ischemic stroke.

**Scheme 1:**
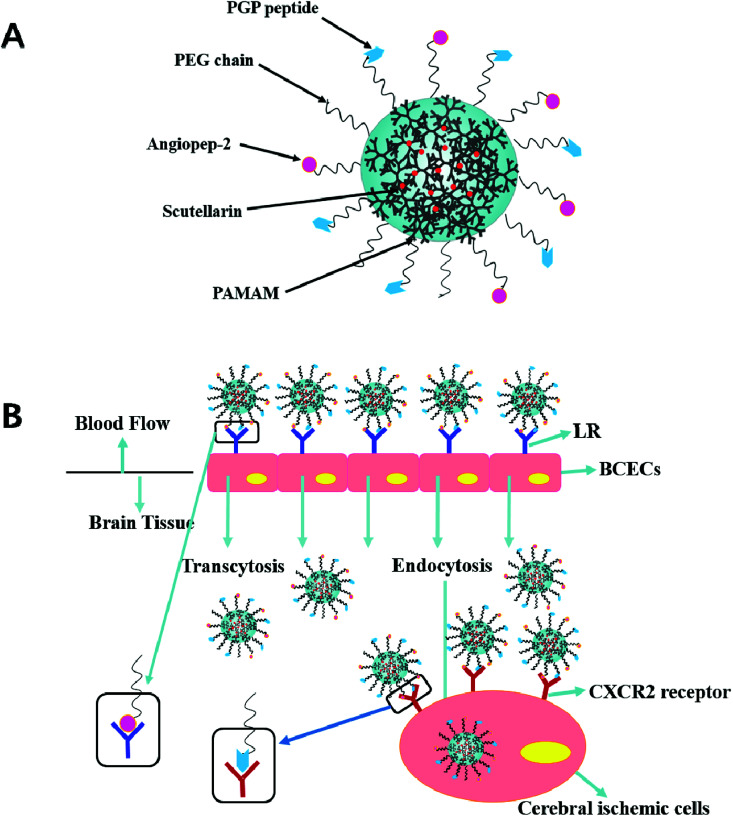
Schematic illustration of dual-targeting ligands functionalized nanoformulation (BBB-penetrating and inflammatory-targeting delivery) *via* low density lipoprotein receptor-mediated and neutrophils-anchored strategy.

In previous studies, the dual targeting strategies have been reported.^21^ Inspired by these findings, we developed novel synergistic dual-ligand Angiopep-2 and PGP modified STA-loaded PEGylated generation-5, hydroxyl-terminated, polyamidoamine dendrimers (PEG-G5.0 PAMAM) NPs (Scheme 1). To the best of our knowledge, this study is the first to report Angiopep-2 and PGP co-functionalized PEG-PAMAM nanocarriers which could transfer therapeutic agent across the BBB. The ultimate goal is to precisely deliver therapeutic compounds to the brain and improve their therapeutic effect against ischemic stroke. To reduce the amine-induced cytotoxicity and achieve prolonged systemic circulation, polyethylene glycol (PEG) was conjugated to the matrix carrier G5.0 PAMAM prior to the self-assembly of the dual ligands functionalized nanoformulation (Angiopep-2-PGP-STA-PEG-PAMAM NPs). The cytotoxicity of this formulation was investigated by MST assay using G5.0 PAMAM NPs and PEG-PAMAM NPs as control groups. We also evaluated its live imaging and transport of NPs across the BBB monolayer.

Although the targeting ligands mediated transport of nano-DDS across BBB have been reported previously,^10–12^ the underlying molecular mechanism of drug-loaded active brain-targeting nanoformulation remains unclear. Our previous work have elucidated the possible mechanism of cationic bovine serum albumin-conjugated nanocarriers on neuronal signaling pathways involved in cerebral ischemia.^3,22^ Therefore, we hypothesize that the protective mechanism of dual-ligand modified nanoformulation on inflammation and neurons injury induced by ischemic stroke is related to modulation of some important pro-inflammatory cytokines and inflammatory signaling pathways. Recently, it has been proved that toll-like receptors like TLR2, TLR4 and TLR5, the myeloid differentiation primary-response protein 88 (MyD88), and the nuclear factor-kappa B (NF-κB) activation as mechanisms linking the pathogenesis of ischemic stroke.^23^ Activation of TLRs causes increased proinflammatory cytokine expression, such as interleukin-17 (IL-17) and interleukin-23 (IL-23). An increasing body of evidence suggests that IL-12p40, IL-17 and IL-23 are important pro-inflammatory cytokines which contributes to neuronal damage in a variety of diseases in CNS, including cerebral ischemia.^24^ TLR2, IL-23 and IL-17 form an axis that leads to increased inflammatory immune responses and neuronal apoptosis. Inhibition of TLR2/IL-23/IL-17 axis significantly suppressed microglia mediated enhancement of neuronal damage.^25^ HMGB1-induced NF-κB activation pathway has gained recognition as a key contributor to the cellular response to neuronal injury in cerebral ischemia. Extracellular HMGB-1 activates its receptor TLR4, thereby promoting the activation of NF-κB.^26^ The TRAM/TRIF pathway is involved in the regulation of other genes in response to TLR2 and TLR4 ligands.^27^ Tumor necrosis factor receptor associated factor 6 (TRAF6) and interleukin-1 receptor-associated kinase-4 (IRAK-4) have been shown to play essential roles in TLR-4-mediated signaling.^28,29^ TRAF6 interacts with the C-terminus of IRAK-1 leading to the activation of downstream signaling pathways like the MAP kinase cascades and the NF-κB inducing pathway. When IRAK-4 is activated, it releases pro-inflammatory cytokines and chemokines (such as NF-κB and TNF-α), which amplify downstream inflammatory mediator cascade.^30^

To test our hypothesis, the effect of synergistic dual-ligand modified nano-carrier on HMGB1/TLRs/MyD88/NF-κB signaling pathways involved in inflammation were evaluated with single-ligand modified STA-loaded nanocarriers and free drug as control. To this end, the mRNA and proteins expressions of major subgroups of HMGB1/TLRs/MyD88/NF-κB signal pathways, including HMGB1, TLR2, TLR4, TLR5, MyD88, TRIF, TRAF6, TRAM, IRAK-4, IκBα, IKKβ and NF-κBp65 were measured by western blotting and real-time quantitative PCR, respectively. The levels of some important proinflammatory cytokines, such as IL-12p40, IL-13, IL-17 and IL-23 in brain tissue homogenates were also determined by enzyme-linked immunosorbent assay (ELISA). The anti-ischemic stroke pharmacological studies and protective effect of dual-ligand modified nano-carrier on neutrophils infiltration and overload of intracellular Ca^2+^ were further assessed.

## 2. Experimental

### 2.1. Animals and cell line

Sprague-Dawley (SD) rats (male, 200 ± 20 g) and Institute of Cancer Research (ICR) mice (male, 20–25 g, Hauschaka, USA) were allowed free access to standard rodent diet and water under controlled conditions (12 h light/dark cycles with relative humidity of 65% ± 5%, 22 ± 1 °C). Hippocampal cells isolated from newborn SD rats were cultured in DMEM medium supplemented with 2% B27 at 37 °C in a 5% CO_2_ humidified atmosphere.

The animal experiments were approved by the Ethical Committee of Medicine School in Yale University. All animals used in this study were handled in accordance with the guidelines of the Principles of Laboratory Animal Care (State Council, revised 2010).

### 2.2. Polymer synthesis and characterization

Hydroxyl-terminated G5.0 PAMAM dendrimer was synthesized according to the situ branch cell method as previously described.^11,31^ The reaction is a two-step iterative process for constructing PAMAM dendrimers possessing either terminal ester or amine groups. This method involves (1) alkylation with methyl acrylate (MA), and (2) amidation with ethylenediamine (EDA). The reactants were repeatedly washed with methanol (once every 2 h) and evaporated under reduced pressure at 40 °C for 8 h to remove the methanol solvent and excess MA. The supernatant was washed with methanol and evaporated under reduced pressure to remove ether and methanol to obtain a higher purity of hydroxyl-terminated G 0.5 PAMAM. The product of hydroxyl-terminated G 0.5 PAMAM was obtained with the yield of 98.7%.

PEGylated PAMAM copolymers were designed and synthesized by conjugating bifunctional NHS-PEG_3400_-MAL to the amine groups of the synthetic hydroxyl-terminated G5.0 PAMAM dendrimers. Briefly, the synthesized hydroxyl-terminated G5.0 PAMAM reacted to NHS-PEG-MAL (MW 3400) with the molar ratio of 1 : 8 in phosphate buffer (PBS, pH 8.0) under magnetic stirring in the dark for 12 h at room temperature. In this stage, the PAMAM primary amine groups react exclusively to terminal NHS groups of bifunctional PEG. The reaction mixture was transferred into an ultrafiltration tube (Millipore, USA, 50 KDa) and centrifuged at 8000 rpm for 30 min in order to separate the unreacted PEG. The yield of MAL-PEG-PAMAM was 57.6% (percentage of the total amount of raw materials, PAMAM and NHS-PEG_3400_-MAL, w/w).

The successful synthesis of hydroxyl-terminated G5.0 PAMAM polymers and MAL-PEG-PAMAM diblock copolymers (dissolved in D_2_O) were demonstrated by ^1^H NMR and ^13^C NMR Bruker AVANCE 500 MHz NMR spectrometer (Switzerland). The infrared spectra were recorded in the spectral range of 400–4000 cm^−1^, using a Nicolet 5700 FT-IR spectrometer with a resolution of 2 cm^−1^. The results on polymer characterization can be found in the ESI (Fig. S2–S11†).

### 2.3. Production and characterization of dual-targeting STA-encapsulated nanoformulation

Angiopep-2 and PGP were covalently linked to PAMAM dendrimer *via* the maleimide function of bifunctional polyethylene glycol (NHS-PEG_3400_-MAL). Briefly, MAL-PEG-PAMAM were dispersed in 0.01 M PBS buffer (pH 7.4), followed by the addition of Angiopep-2 and PGP in the same solvent at the molar ratio of 1 : 1 (maleimide : Angiopep-2) and 1 : 2 (maleimide : PGP), respectively. The conjugation of Angiopep-2 and PGP to the NPs loaded with STA was performed under gentle magnetic stirring. After 12 h of reaction at room temperature, the small molecular substances were separated from the conjugate on a Sephadex G-25 fine column eluted with PBS (pH 7.4). The macromolecular fractions of Angiopep-2-PGP-PEG-PAMAM NPs were collected and ultrafiltrated, then nano-dendrimer solution was obtained.

Angiopep-2-PGP-PEG-PAMAM NPs was dissolved in deionized water, and the proper amount of STA was added under magnetic stirring. The reaction solution was stirred for 3 h in the dark, and filtered with 0.45 μm microporous filtering film. Blank dual-ligand modified nanocarriers were prepared by the same method except that STA was not added.

^1^H NMR spectra of Angiopep-2-PGP-STA-PEG-PAMAM NPs (in D_2_O) were recorded on Bruker AVANCE 500 MHz NMR spectrometer (Switzerland). The morphology of Angiopep-2-PGP-STA-PEG-PAMAM NPs was observed by transmission electron microscope (TEM, JEOL, Japan) and scanning electron microscope (SEM, S-4800, Hitachi, Japan). Size of the particle and zeta potential of Angiopep-2-PGP-STA-PEG-PAMAM NPs were measured by dynamic light scattering with a NICOMP™ 380/ZLS zeta potential/particle size system (Santa Barbara, USA). The nanoformulations were diluted with an appropriate volume of distilled water before zeta potential measurement. The coupling efficiency of Angiopep-2 and PGP to PAMAM NPs was determined as described previously.^32^

The drug loading efficiency (LE) and entrapment efficiency (EE) of Angiopep-2-PGP-STA-PEG-PAMAM NPs was assayed by UltiMate® 3000 DGLC HPLC. LE and EE were calculated by the following equations:1
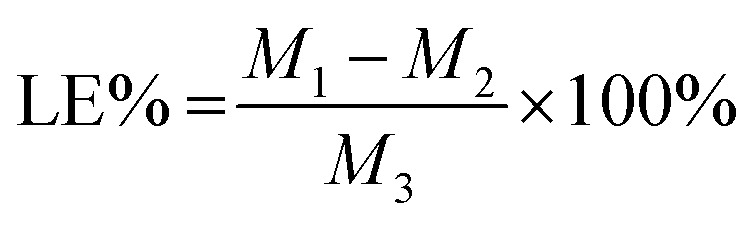
2
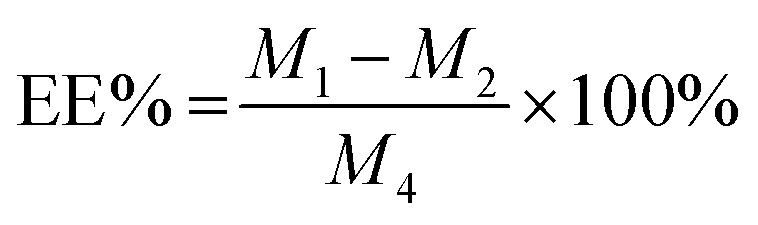
where *M*_1_ is the total amount of STA added into nano-DDS; *M*_2_ is the free unloaded STA in Angiopep-2-PGP-STA-PEG-PAMAM suspension; *M*_3_ is total amount of Angiopep-2-PGP-STA-PEG-PAMAM NPs; *M*_4_ is the total amount of free STA.

### 2.4. *In vivo* imaging analysis and *ex vivo* distribution

The *in vivo* imaging analysis *ex vivo* distribution of different NPs were studied under live imaging system, using DiR as the fluorescent probe.^33,34^ Briefly, MAL-PEG-PAMAM (14.5 mg) were dispersed in 5 ml 0.01 M PBS buffer (pH 7.4), followed by the addition of Angiopep-2 and PGP at the molar ratios of 1 : 1 (maleimide : Angiopep-2) and 1 : 2 (maleimide : PGP), respectively. After 24 h of reaction at room temperature, DiR fluorescent dye (1 mg) dissolved in 0.1 ml DMSO was added to the same solvent. The solution was stirred overnight in the dark at room temperature. The DiR-loaded single targeting ligand modified nanocarriers and DiR-loaded unmodified NPs were prepared in the similar method as described above.

DiR-loaded Angiopep-2-PEG-PAMAM NPs, DiR-loaded PGP-PEG-PAMAM NPs and DiR-loaded Angiopep-2-PGP-PEG-PAMAM NPs were injected through the tail vein of ICR mice at a dose of 10 μg of DiR, respectively. Then, the mice were sacrificed by injection of chloral hydrate *via* tail vein. The *in vivo* imaging was performed at 2 h post-injection using BRUKER IS4000MM PRO image station system (Bruker, BioSpin, Corporation, USA). The DiR-loaded PAMAM NPs and DiR-loaded PEG-PAMAM NPs served as control, respectively.

After that, the brains and other major organs (including heart, liver, lung, spleen and kidney) were harvested, and the *ex vivo* imaging of the organs was also captured. Blood was cleared prior to *ex vivo* fluorescent visualization.

### 2.5. *In vivo* penetration across BBB

To determine *in vivo* penetration of dual-ligand functionalized nanoformulation across BBB into the brain, the efficiency of different nanoparticles into the brain was evaluated as previous studies.^3^ Briefly, different nanoparticles loaded with coumarin-6 dye (35 mg kg^−1^) were infused through the tail vein of rats at the time of reperfusion, respectively. After administration of coumarin-6-loaded different NPs 2 h, the rats were sacrificed and the brains were collected after perfusion with normal saline and 4% paraformaldehyde respectively. After anhydration, the brains were cut into 5 μm slices by frozen section. Then, the brain slices were examined under a laser scanning confocal microscope (Zeiss LSM 510; Jena, Germany) using a fluorescein isothiocyanate filter (excitation/emission, 495/520 nm).

### 2.6. Transport of NPs across the BBB monolayer

The BBB is localized at the interface between blood and cerebral tissue.^35^ BCECs are the most important component to maintain the function of BBB. It is an attractive candidate for BCECs as a model of the BBB because of their rapid growth, maintenance of BBB characteristics over repeated passages, formation of functional barriers, and amenability to numerous molecular interventions. So it was widely used as BBB model *in vitro* for evaluating the effect of drug nano-DDS across BBB.^12,35^

In order to elucidate the cellular uptake mechanism of dual-ligand functionalized nanoformulation across the BBB, BCECs BBB monolayers were established as reported previously.^35^ Briefly, BCECs were seeded into the 24-well Transwell upper chambers at a density of 5 × 10^4^ cells per well, and cultured for about 1 week. The culture medium was changed every 2 days. Before starting the experiment, the integrity of the BCECs monolayer was assessed by measuring the transendothelial electrical resistance (TEER) using Millicell ERS (Millipore, Billerica, MA). Only the BCECs monolayers with TEER over 200 Ω cm^2^ were used for transcytosis BBB study.

For qualitative studies, the abilities of cellular internalization of different NPs were visualized under a fluorescent microscope, using coumarin-6 as the fluorescent probe.^36^ The BCECs were incubated with fresh complete DMEM containing different nanoparticles with an equivalent concentration of coumarin-6 at 5 ng ml^−1^ for 1 h, respectively. After the incubation, cells were washed three times with cold PBS (pH 7.4), and fixed with 4% formaldehyde for 15 min. The cells were visualized using a fluorescent microscope (Imager A1, Zeiss, Germany).

We also quantitatively evaluate the transport efficiency of different coumarin-6 loaded NPs uptake by BCECs, previous published reports.^37^ The BCECs were incubated with 100 μg ml^−1^ coumarin-6-loaded NPs for 1 h at 37 °C. After the incubation, cells were washed with PBS, fixed with 4% formaldehyde for 15 min, stained with 2 μg ml^−1^ Hoechst 33258 at room temperature for 10 min away from light. The fluorescence intensity of cells was measured by a flow cytometer (Cytomics™ FC 500; Beckman Coulter, Miami, Florida).

### 2.7. Histological study

For histology, brain coronal sections were fixed in 4% paraformaldehyde, then dehydrated, subsequently embedded in paraffin, and finally sectioned in 5–7 μm thick sections. The samples were stained with Hematoxylin–Eosin (HE) solution Prepared as previously described,^3^ and examined with a microscopy (200×, Leica, Germany).

All brain sections were qualitatively examined by two experienced pathologists in a blinded fashion.

### 2.8. Determination of cerebral myeloperoxidase (MPO) activity and inflammatory cytokine levels

MPO activity was measured at 24 h after cerebral ischemia. MPO activity was used as an indicator of neutrophils infiltration in cerebral ischemia process.^3^ PGP can be probably used as a specific neutrophil anchoring peptide for brain-targeting delivery. We determined the MPO activity in order to validate and compare the brain-targeting efficiency of single PGP ligand-mediated STA-encapsulated nanoformulation with dual-ligand mediated STA-encapsulated nanoformulation, and further evaluate their inhibitory effects on neutrophil infiltration in ischemic stroke. MPO activity was determined with the *O*-dianisidine method by absorbance at 460 nm. The brain tissues of each group were homogenized in ice-cold physiological saline at the final concentration of 10% (w/v). The quantitative procedure was carried out according to the recommendations of detection kit. MPO was expressed in units per gram of tissue.

At 24 h of reperfusion, rats in each group were sacrificed and the brain tissues were homogenized in ice-cold physiological saline at the final concentration of 10% (w/v). The concentrations of IL-12p40, IL-13, IL-17 and IL-23 levels in brain tissue homogenates were quantified using ELISA kits according to the manufacturer's instructions.

### 2.9. Neuronal cell culture and quantification of [Ca^2+^]i in hippocampal neurons

Primary neuronal cultures were prepared from newborn SD rats within 24 h. The tissue was digested with 0.25% trypsin at 37 °C for 30 min. Hippocampal cells were isolated and washed with D-Hank's solution three times in sterile conditions. Cells were seeded at a density of 1.5 × 10^6^ cells per cm^2^ in poly-l-lysine coated plates and maintained in a humidified incubator (37 °C, 5% CO_2_). Neurons were cultured in DMEM (Gibco, CA, USA) that had been supplemented with 2% B27 (Gibco, CA, USA), 2 mM glutamine and 100 U per ml penicillin per streptomycin. Half of the cell culture media was changed twice a week. For oxygen/glucose deprivation (OGD), the cultures were washed twice in glucose-free balanced salt solution (BSS, pH 7.4) containing: 130 mM NaCl, 5.5 mM KCl, 1.8 mM CaCl_2_, 1.0 mM MgCl_2_, 20 mM HEPES, and incubated in oxygen- and glucose-free BSS (pretreated by gassing 95% N_2_ and 5% CO_2_) in an incubator containing 95% N_2_ and 5% CO_2_ at 37 °C for 2 h. After OGD, cultures were replaced into neurobasal medium and maintained in a CO_2_ incubator for 24 h as OGD/reperfusion. The dual-targeting drug-loaded nanoformulation (100 μM), Angiopep-2-STA-PEG-PAMAM NPs (100 μM), PGP-STA-PEG-PAMAM NPs (100 μM), vehicle controls and STA solution (100 μM) were added to the culture 24 h before the reperfusion, respectively.

Cells were washed three times with Hank's solution, and then loaded with 10 μ mol l^−1^ Fluo-3/AM for 30 min at 37 °C. After 30 min at 37 °C the cells rinsed three times with Hank's solution to remove the extracellular Fluo-3/AM. The fluorescence intensity of [Ca^2+^]i in hippocampal neurons was determined by laser scanning confocal microscopy (Zeiss, LSM510, Germany). Total images were scanned and the data were stored in disks for analyzing. The intracellular calcium concentration was calculated according to the fluorescence intensity.

### 2.10. Western blotting

Western blotting was performed as described previously with some modification.^38^ Brain homogenates were lysed in ice-cold lysis buffer (50 mM Tris–HCl, pH 7.4, 150 mM NaCl, 1 mM EDTA, 1% SDS, 0.25% NaTDC, 1 mM PMSF, 1 mg l^−1^ aprotinin, 1 mg ml^−1^ leupeptin, 1 mM DTT and 1% Triton100). The lysates were then separated by 10% SDS-polyacrylamide gel electrophoresis and electrophoretically transferred onto polyvinylidene fluoride membrane (Millipore, MA, USA). The membranes were blocked with 5% skim milk dissolved in Tween-20/Tris-buffered saline (TTBS) for 2 h at room temperature. Next, the membrane was incubated with antibodies against HMGB1, TLR2, TLR4, TLR5, MyD88, IRAK-4, TRAM, TRAF6, NF-κBp65, IκBα, p-IκBα and IKKβ (1 : 500, cell signaling) in TTBS containing 5% skim milk overnight at 4 °C. Protein expression was detected with ECL western blotting detection reagent. GAPDH was used for normalization.

### 2.11. Real-time PCR

Total RNA was isolated from brain tissue using Trizol reagent (Invitrogen, Carlsbad CA, USA), and cDNA was prepared from 2 mg of total RNA according to the protocol of the kit. Real-time quantitative PCR was performed by use of an ABI Prism 9700 sequence detector using 2.5 ml (1 mg ml^−1^) cDNA, 12.5 ml SYBR Green PCR Master Mix (2×), 2.5 ml primer pair mix (5 pmol each primer), and water to a 25 ml final volume. The specific primers were designed by Primer Premier 5.0 software (Molecular Biology Insights, USA). The primer sequences used in PCR amplification are shown in (ESI Table S1†). Real-time PCR was used to analyze the levels of HMGB1, TLR2, TLR4, TLR5, MyD88, NF-κBp65, IκBα, IKKβ, TRAF6, IRAK-4, TRAM and TRIF mRNA at 24 h after MCAO (*n* = 6 per group). Results were analyzed by use of Sequence Detection Software (Applied Biosystems, Foster City, Calif), and the target gene expressions of mRNA was normalized to GAPDH (18 S rRNA endogenous control).

### 2.12. Statistics

The experiments were performed in triplicates. The results were presented as mean ± standard deviation (mean ± SD). Statistically significant differences by Student's *t*-test when compared to the corresponding value of the control. Differences were considered significant when *p* < 0.05. Statistical analysis were performed using SPSS 19.0 software for Windows (IBM Corporation, USA).

## 3. Results and discussion

### 3.1. Characterization of dual-targeting nanoformulation

We firstly synthesized hydroxyl-terminated G5.0 PAMAM dendrimer (purity > 98%) as the matrix nanocarrier by *in situ* branch cell method as reported previously.^11,31^ The chemical structure of G5.0 PAMAM was confirmed by ^1^H NMR, ^13^C NMR and infrared spectrogram (IR) (Fig. S2–S4†). The successful synthesis of hydroxyl-terminated G5.0 PAMAM polymers was demonstrated by multiple characteristic peaks belonged to the branching units of PAMAM. In ^1^H NMR spectrum (Fig. S2†), the multiple peaks between 2.3 and 3.2 ppm belonged to the methylene protons of branching units of hydroxyl-terminated G5.0 PAMAM. The proton of terminal amino group presented the corresponding peak at 2.327 ppm (Fig. S2†). The ^13^C NMR data (Fig. S3†) shows a single peak at 174.96/174.51 ppm attributed to C=O group in PAMAM segment. Significant FT-IR spectral changes were detected in the region of 1000–4000 cm^−1^ containing –NH_2_, –CONH, –CH_2_–, C–N, tertiary amine and other functional characteristic groups (Fig. S4†), which is consistent with the theoretical structure of hydroxyl-terminated G5.0 PAMAM. The peak at 1638.01 cm^−1^ pertained to the amide bond, the peak at 1560.34 cm^−1^ is the stretching vibration of N–H bond and C–H bond in the amide bond; the peak at 1466.52 cm^−1^ corresponded to the bending vibration of –CH_2_. The peaks at 1198.74 cm^−1^ and 1127.36 cm^−1^ are the stretching vibrations of primary and tertiary amines, respectively.

Despite the enormous advantages of nanoformulations for treatment of ischemic stroke, a fundamental concern that impedes their clinical application is the shuttling out of the nano cargos from systemic circulation by the reteculoendothelial system (RES).^22,39^ To address this issue, we modified hydroxyl-terminated G5.0 PAMAM dendrimer with bi-functional PEG due to its increased *in vivo* stability, with sustained levels in circulation and evasion of RES. Some reports have demonstrated that PEG chains was functionalized to the surface of the NP to prolong its half-life in circulation and minimize opsonization which leads to the non-specific uptake in normal tissue.^37,40^

PEGylated PAMAM nanocarriers were designed and synthesized by conjugating bi-functional NHS-PEG_3400_-MAL to the amine groups of the synthetic hydroxyl-terminated G5.0 PAMAM dendrimers. The chemical structures of MAL-PEG-PAMAM were confirmed by ^1^H NMR spectra, ^13^C NMR spectra, Thin-Layer Chromatography (TLC) and infrared spectrogram (IR) (Fig. S5–S8†). The successful synthesis of MAL-PEG-PAMAM diblock copolymers was confirmed by the appearance of a signal at 3.578 ppm (^1^H NMR) that corresponded to the methylene protons of NHS-PEG-MAL (Fig. S5†).^41^ Furthermore, the repeat units of PEG presented a sharp peak at 3.7 ppm (^1^H NMR, Fig. S5†), showing that the MAL group had reacted with the thiol group of Angiopep-2.^42^ The methene group (–CH_2_–) of PEG segment appeared at 69.68 ppm (^13^C NMR, Fig. S6†). As shown in FT-IR spectrum (Fig. S8†), MAL-PEG-PAMAM showed its unique characteristic peaks in the region from 1400 cm^−1^ to 1100 cm^−1^. Furthermore, stretching peak of primary amines near 1198 cm^−1^ attributed to G5.0 PAMAM disappeared, and a new peak was observed at 3429.32 cm^−1^, which was just the primary amino peak of G5.0 PAMAM present at 3288.68 cm^−1^ (Fig. S8†) and red-shifted to 3439.32 cm^−1^ in FT-IR spectrum of MAL-PEG-PAMAM (Fig. S8†). The peak at 2918.13 cm^−1^ was the stretching vibration peak of –CH_2_ belonged to MAL-PEG-PAMAM, and the peak at 1101.92 cm^−1^ is the stretching vibration peak of C–O–C in PEG. So it can be concluded that NHS-PEG_3400_-MAL reacted with the primary amino groups on the surface of hydroxyl-terminated G5.0 PAMAM.

Finally, Angiopep-2 and PGP targeting peptides were covalently linked conjugated to NHS-PEG-MAL_3400_ by the reaction between the maleimide group of PEG and the thiol group of Angiopep-2 and PGP. ^1^H NMR and dynamic light scattering zeta potential/particle size system were used to analyze the exact chemical and physical characteristics of dual-ligand modified nanocarriers (Fig. S9–S11†). Significant spectral changes are detected in the region 6.177–8.322 ppm (Fig. S9†) assigned to the corresponding peaks of Angiopep-2. The repeat units of PEG presented a sharp peak at 3.7 ppm, showing that the MAL group had reacted with the thiol group of Angiopep-2. The peak around 7.6 ppm and 6.8 ppm represented the protons in PGP histidine units, indicating the existence of PGP peptide (Fig. S9†). Thus, the ^1^H NMR spectra (Fig. S9†) results proved the successful synthesis of Angiopep-2-PGP-PEG-PAMAM. The coupling efficiency of Angiopep-2 and PGP to MAL-PEG-PAMAM was around 36.4% and 39.6%, respectively.

**Fig. 1:**
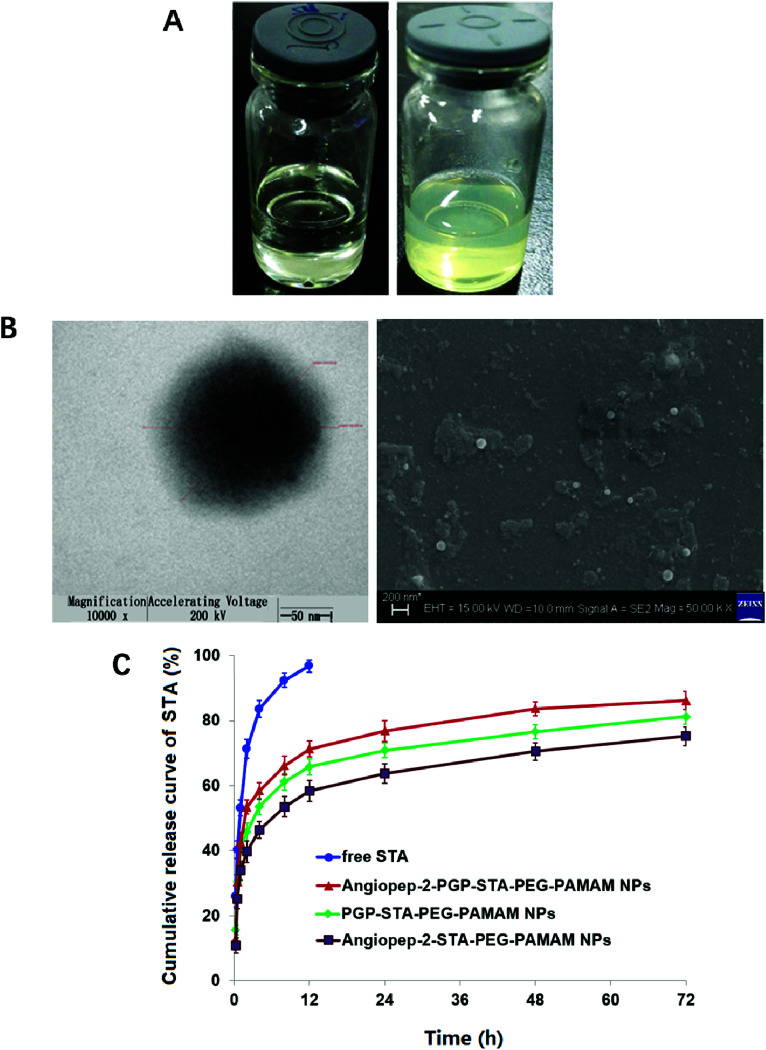
Characterization of STA-encapsulated Angiopep-2-PGP-PEG-PAMAM NPs. Photograph of blank Angiopep-2-PGP-PEG-PAMAM NPS dispersion and STA-encapsulated Angiopep-2-PGP-PEG-PAMAM NPs dispersion containing 58.97 ± 0.6% w/w scutellarin (A); transmission electron microscope (TEM) and scanning electron microscope (SEM) image of Angiopep-2-PGP-STA-PEG-PAMAM NPs (B) and the cumulative release curve of Angiopep-2-PGP-STA-PEG-PAMAM NPs (C).

Blank Angiopep-2-PGP-PEG-PAMAM NPs exhibited a translucent nanoscale colloid solution, while STA-encapsulated Angiopep-2-PGP-PEG-PAMAM NPs showed pale yellow nanoscale colloid solution (Fig. 1A). TEM and SEM images also revealed the successful preparation of Angiopep-2-PGP-STA-PEG-PAMAM NPs with regularly spherical-shaped structures (Fig. 1B). Dynamic light scattering (DLS) revealed Angiopep-2-PGP-STA-PEG-PAMAM NPs with average diameter equal to 171 ± 22 nm (Fig. 1B and S10†), and a low polydispersity index (PDI) (0.31 ± 0.05). The *ζ*-potential of Angiopep-2-PGP-STA-PEG-PAMAM NPs was determined to be 6.4 mV (Fig. S11 and S14†). Angiopep-2-PGP-STA-PEG-PAMAM NPs showed higher drug loading capacity (58.97 ± 0.6% of free drug, w/w) compared to conventional nanoformulations of STA (usually about 15%, w/w),^43^ and the encapsulation efficiency was 80.61 ± 3.4%. High drug loading capacity will greatly facilitate therapeutic efficiency of ischemic stroke and reduce excipient-associated toxicities.

### 3.2. A safe and stable nanoformulation of STA

The cell viability of BCECs after incubation with blank G5.0 PAMAM NPs, PEG-PAMAM NPs, Angiopep-2-PEG-PAMAM NPs, PGP-PEG-PAMAM NPs and Angiopep-2-PGP-PEG-PAMAM NPs for 48 h was displayed in Fig. S12.† G5.0 PAMAM dendrimer was indeed cytotoxic against BCECs when its concentration exceeded 1 μmol l^−1^, and the cell viability of BCECs was less than 40%. It has been demonstrated that amine-terminated PAMAM dendrimers generally exhibited generation dependent toxicity.^10^ In the present study, we found that modification of bifunctional NHS-PEG_3400_-MAL can remarkably reduce the cytotoxicity of G5.0 PAMAM due to the fact that more than 70% cells are still alive at the experimental concentration range, even when the concentrations of blank PEG-PAMAM NPs, blank Angiopep-2-PEG-PAMAM NPs, blank PGP-PEG-PAMAM MPs and Angiopep-2-PGP-PEG-PAMAM NPs exceeded 1000 μmol l^−1^. Besides, there were no significant differences in cytotoxicity between PEG-PAMAM, single-ligand modified nanocarriers and synergistic dual-targeting nanoformulation at experimental concentrations. This result suggested that the dual-targeting nanoformulation is safety.

Colloidal stability of drug-loaded nanocarriers is crucial for their long-term storage and transportation.^44^ As shown in Fig. S13 and S14,† the average size and zeta potential of Angiopep-2-PGP-STA-PEG-PAMAM NPs at 0, 5, 10, 15 and 30 days gradually declines over three-months of storage in solution, reflecting surface-hydrolysis. Interestingly, at all time points tested, the size of NPs is smaller in alkaline medium (pH = 9.0) than acidic medium (pH = 5.0), consistent with the faster degradation rate of polyesters in basic environments. Nevertheless, this slight difference in size did not significantly affect drug release profiles in the first 3 days (Fig. 1C). No sign of aggregation is observed in the experimental conditions.

### 3.3. *In vitro* sustained release of STA nanoformulation

*In vitro* drug release results suggest sustained STA release from two single-ligand modified nanoformulation and the dual-targeting nanoformulation beyond 72 h (Fig. 1C). As shown in this experiment, 93.5% of free STA are detected outside the dialysis tube within 8 h, and 98.8% are detected at 12 h. In contrast, less than 70% of STA are released from three nanoformulations at 12 h. After this initial burst-release phase, the dual-targeting nanoformulation showed sustained drug release property in the next 60 h with 15.4% cumulative release, with a mean release rate of 0.26% per hour, which was much slower than that of free STA (8.23% per hour). We speculate that the burst-release phase of STA from the nanoparticles reflects the dissociation of surface-bound drug from NP surfaces, and that the slow and sustained second release phase is due to the erosion of the polymer matrix. Specially, the entire process of STA release from dual-targeting nanoformulation best fits the Korsmeyer–Peppas model (*r*^2^ = 0.9834), followed by zero order (*r*^2^ = 0.9681), Higuchi (*r*^2^ = 0.9476), Hixson–Crowell (*r*^2^ = 0.8149), and first order (*r*^2^ = 0.7457). The magnitude of the release exponent “*n*” in Korsmeyer–Peppas's model indicates that the release mechanism was super case II transport, suggesting that the drug release was due to plasticization, swelling and relaxation of the polymeric matrix.

### 3.4. Nanoformulation improved STA pharmacokinetics and altered its biodistribution

The limited clinical outcomes of commercially conventional STA formulations might be attributed to low drug loading capacity,^43^ fast metabolic rate^5^ and poor bioavailability and permeability across the blood–brain barrier (BBB).^6^ To exert its function, we hypothesize that the pharmacokinetic profile of STA can be ameliorated by loading it into a biodegradable dual-targeting brain delivery nanoformulation, thereby improving its therapeutic outcomes.

To evaluate the *in vivo* performance of the dual-ligand modified nanoformulation, the systemic pharmacokinetics in cerebral ischemia rats were studied with single-ligand modified drug-loaded nanocarriers, unPEGylated drug-loaded PAMAM NPs and free STA as the control. The plasma drug concentration–time curves of STA after the injection of five formulations were exhibited in Fig. S15A.† The main pharmacokinetics parameters were calculated and summarized in Table. S2.† As shown in Fig. S15A,† free STA was exponentially cleared from blood, and was almost not detected after 6 h due to its poor stability and short half-life, which could be easily phagocytosed by the reticuloendothelial system (RES).^5,6^ UnPEGylated drug-loaded PAMAM NPs also demonstrated a short half-life in plasma, indicating rapid clearance duo to without PEGylated modification. By contrast, both single-ligand modified drug-loaded PEGylated nanoformulations and dual-targeting drug-loaded PEGylated nanoformulation exhibited similar initial phase of rapid decrease in plasma concentration after injection of 45 min, while much slower decline after 50 min post dose, and showed significantly prolonged retention in blood, which could still keep higher drug concentrations in plasma 12 h after intravenous administration. Especially, the plasma drug concentration of the dual-targeting drug-loaded PEGylated nanoformulation and single-ligand modified drug-loaded PEGylated nanoformulations could be measured in plasma even after 20 h. In addition to improving the stability of drug-loaded nanoparticles, the PEG chains on the outer surface of nanocarriers played an important role in avoiding identification and phagocytosis by RES.^45^ As displayed main pharmacokinetics parameters of Angiopep-2-PGP-STA-PEG-PAMAM NPs, Angiopep-2-STA-PEG-PAMAM NPs, PGP-STA-PEG-PAMAM NPs, STA-PAMAM NPs and free STA in Table. S2,† the half-life (*t*_1/2β_) and the mean residence time (MRT) of Angiopep-2-PGP-STA-PEG-PAMAM NPs was significantly extended 5.3 times and 6.7 times compared with free STA, respectively. The *t*_1/2β_ and MRT of the dual-targeting drug-loaded nanoformulation were also higher than that of single-ligand modified drug-loaded nanocarriers (Table. S2†), whereas, the clearance ratio (CLs) of Angiopep-2-PGP-STA-PEG-PAMAM NPs decreased 6.4 times than that of free STA and unPEGylated drug-loaded PAMAM NPs. These results suggested the blood circulation times of the dual-targeting nano-drug delivery system and single-ligand modified drug-loaded nanocarriers were all markedly prolonged by PEGylation. PEGylation can both improve the water and lipid solubility of drugs.^46^ Improved water solubility can enhance the bioavailability of STA in nanoformulations (Table. S2†). In comparison to free STA and unPEGylated drug-loaded PAMAM NPs, Angiopep-2-PGP-STA-PEG-PAMAM NPs achieved the significantly higher AUC_0–∞_ (*p* < 0.01) with a relative bioavailability of 318%. Furthermore, improved lipid solubility may facilitate the penetration of STA through the BBB. In addition, with PEG modification, the dendrimer nanoparticles can also improve the possibility of binding between dual-targeting ligands (Angiopep-2 and PGP) and their receptors in the BBB and ischemic inflammatory site, which contributes to the higher precise brain-targeting uptake.^13,47^

The distribution profiles of free STA, STA-encapsulated PEG-PAMAM NPs, Angiopep-2-STA-PEG-PAMAM NPs, PGP-STA-PEG-PAMAM NPs and Angiopep-2-PGP-STA-PEG-PAMAM NPs into plasma and various organs (heart, liver, spleen, lung, kidney and brain) at 6 h after intravenous administration was shown in Fig. S15B.† Free drug was seldom accumulated in plasma and mainly distributed to liver, spleen and kidney, respectively. It is well known that liver and kidney are main organs of drug metabolism.^12^ This result indicated that free STA could be quickly phagocytized in RES and eliminated through liver and kidney. However, Angiopep-2-STA-PEG-PAMAM NPs, PGP-STA-PEG-PAMAM NPs and Angiopep-2-PGP-STA-PEG-PAMAM NPs exhibited significant increase of drug accumulation in plasma and brain compared to STA-encapsulated PEG-PAMAM NPs and free STA. Our previous work demonstrated that drug encapsulated in nanoparticles can be protected from metabolizing enzyme in the liver and phagocytosis of the reticuloendothelial system.^3,22^ The results provided the evidence that STA-encapsulated PEG-PAMAM NPs, Angiopep-2-STA-PEG-PAMAM NPs, PGP-STA-PEG-PAMAM NPs and Angiopep-2-PGP-STA-PEG-PAMAM NPs could prolong circulation time and reduce drug metabolism due to PEGylation. In comparison to free STA, PEG-PAMAM-STA NPs can increase drug accumulation in the brain (*p* < 0.05). By contrast, Angiopep-2-STA-PEG-PAMAM NPs and PGP-STA-PEG-PAMAM NPs showed higher drug accumulation in the brain than that of unmodified STA-encapsulated PEG-PAMAM NPs (*p* < 0.01). Specially, Angiopep-2-PGP-STA-PEG-PAMAM NPs exhibited significant increase of brain targeting delivery of STA compared with unmodified STA-encapsulated PEG-PAMAM NPs and any single-ligand modified STA-encapsulated nanocarriers (*p* < 0.01), due to dual-ligand mediated better permeability across the BBB and further target inflammatory sites of ischemic brain.

### 3.5. Dual-ligand functionalization increases brain targeting the nanoparticles

**Fig. 2:**
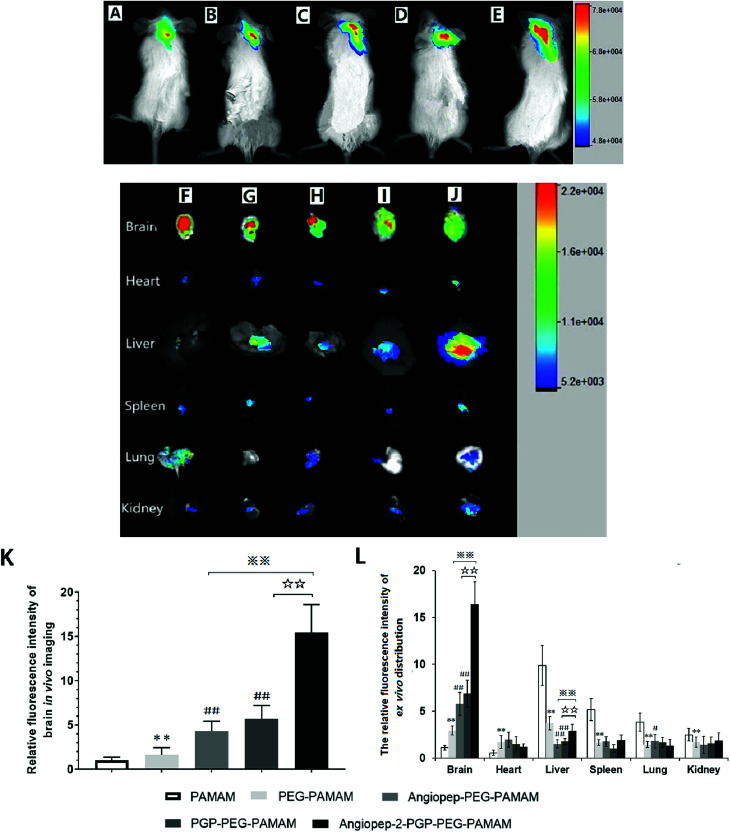
Representative *in vivo* imaging and *ex vivo* Biodistribution. *In vivo* fluorescence images of animals at 2 h after intravenous injection of DiR-loaded PAMAM NPs (A), DiR-loaded PEG-PAMAM NPs (B), DiR-loaded Angiopep-2-PEG-PAMAM NPs (C) DiR-loaded PGP-PEG-PAMAM NPs (D) and DiR-loaded Angiopep-2-PGP-PEG-PAMAM NPs (E). *Ex vivo* fluorescence images of organs harvested 120 min after intravenous injection of DiR-labeled PAMAM NPs (J), DiR-loaded PEG-PAMAM NPs (I), DiR-loaded Angiopep-2-PEG-PAMAM NPs (H) DiR-loaded PGP-PEG-PAMAM NPs (G) and DiR-loaded Angiopep-2-PGP-PEG-PAMAM NPs (F). Quantitative analysis of the relative fluorescent intensity of the different DiR-loaded nanoformulations in brain live imaging (K); Quantitative fluorescence intensity of *ex vivo* biodistribution (brain and other different organs) (L). Statistically significant differences by Student's *t*-test when compared to the corresponding value of the control. Data represented mean ± SD (*n* = 5). **p < 0.01 *vs.* DiR-loaded PAMAM NPs; ##*p* < 0.01 *vs.* DiR-loaded PEG-PAMAM NPs; ☆☆ *p* < 0.01 *vs.* DiR-loaded PGP-PEG-PAMAM NPs; ※※*p* < 0.01 *vs.* DiR-loaded Angiopep-2-PEG-PAMAM NPs.

To validate the active targeting ability of dual-ligand modified nano-drug delivery system to the brain, *in vivo* imaging analysis and *ex vivo* organ distribution were performed in cerebral ischemia ICR mice. UnPEGylated PAMAM NPs, PEG-PAMAM NPs and single-ligand modified nanocarriers were used as control in all evaluations. The cerebral ischemia ICR mice were injected with PAMAM NPs, PEG-PAMAM NPs, Angiopep-2-PEG-PAMAM NPs, PGP-PEG-PAMAM NPs and Angiopep-2-PGP-PEG-PAMAM NPs loading a fluorescent probe DiR at a dose of 10 μg, respectively. *In vivo* fluorescent images were prepared at 2 h after injection. As shown in Fig. 2A and B, weak fluorescent signals were exhibited in the brains of control animals treated with DiR-loaded PAMAM NPs and DiR-loaded PEG-PAMAM NPs, because without the help of active-targeting ligand, NPs cannot penetrate the BBB spontaneously. By contrast, single-ligand modified DiR-loaded nanocarriers, such as DiR-loaded Angiopep-2-PEG-PAMAM NPs (Fig. 2C) and DiR-loaded PGP-PEG-PAMAM NPs (Fig. 2D) accumulated relatively stronger in the ischemic brain of ICR mice than that of DiR-loaded PAMAM NPs (Fig. 2A) and DiR-loaded PEG-PAMAM NPs (Fig. 2B, *p* < 0.05). The relative fluorescence intensity of DiR-loaded Angiopep-2-PEG-PAMAM NPs were 5.3-fold and 2.8-fold as that for DiR-loaded PAMAM NPs and DiR-loaded PEG-PAMAM NPs, respectively. Furthermore, the relative fluorescence intensity of DiR-loaded PGP-PEG-PAMAM NPs (Fig. 2D) was 6.7-fold and 3.4-fold as that for DiR-loaded PAMAM NPs and DiR-loaded PEG-PAMAM NPs, respectively. Notably, the accumulation of DiR-loaded dual-targeting nanoformulation (Fig. 2E) in the ischemic brain of ICR mice was much higher than that of DiR-loaded Angiopep-2-PEG-PAMAM NPs and DiR-loaded PGP-PEG-PAMAM NPs. The relative fluorescence intensity of DiR-loaded dual-targeting nanoformulation were 15.6-fold and 5.5-fold as that for DiR-loaded PAMAM NPs and DiR-loaded PEG-PAMAM NPs, respectively. We could conclude that the dual-targeting nanoformulation could markedly enhance the brain targeting effect compared with single-ligand modified nanocarriers due to the stepwise-targeting mechanism by the combined modification of Angiopep-2 and PGP.

**Fig. 3:**
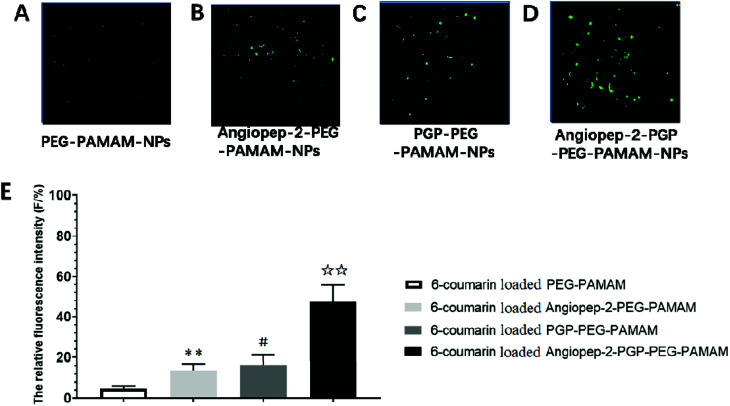
*In vivo* evaluation of BBB permeability. The brain uptake study of different 6-coumarin loaded NPs in brain slices by laser scanning confocal microscope (A–D). The relative fluorescence intensity (*F*/%) of coumarin-6-loaded PEG-PAMAM NPs, coumarin-6-loaded Angiopep-2-PEG-PAMAM NPs, coumarin-6-loaded PGP-PEG-PAMAM NPs and coumarin-6-loaded Angiopep-2-PGP-PEG-PAMAM (E). Statistically significant differences by Student's *t*-test when compared to the corresponding value of the control. Data represented mean ± SD (standard deviation) (*n* = 5). **p < 0.01 *vs.* coumarin-6-loaded PEG-PAMAM NPs; #*p* < 0.05 *vs.* coumarin-6-loaded Angiopep-2-PEG-PAMAM NPs; ☆☆*p* < 0.01 *vs.* coumarin-6-loaded PGP-PEG-PAMAM NPs.

The *ex vivo* organ distribution of different NPs was also studied in cerebral ischemic ICR mice (Fig. 2F–J). As shown in Fig. 3I and J, the unmodified nanocarriers, including DiR-loaded PAMAM NPs and DiR-loaded PEG-PAMAM NPs exhibited weaker fluorescent intensity in the dissected brain. In comparison to the unmodified nanocarriers, single-ligand modified DiR-loaded nanocarriers, such as DiR-loaded Angiopep-2-PEG-PAMAM NPs (Fig. 2H) and DiR-loaded PGP-PEG-PAMAM NPs (Fig. 2G) showed a much higher fluorescent accumulation in the brain tissue (*p* < 0.01). Notably, the enhanced fluorescent intensity of DiR-loaded Angiopep-2-PGP-PEG-PAMAM NPs (Fig. 2F) was observed at remarkably higher level in the dissected brain than that of single-ligand modified DiR-loaded nanocarriers (Fig. 2H and G, *p* < 0.01). With dual ligand-mediated brain-targeting transcytosis, Angiopep-2-PGP-PEG-PAMAM NPs showed around 3-fold and 12-fold higher brain accumulation in ICR mice than that of single-ligand modified nanocarriers and PEG-PAMAM NPs at 120 min post-injection, respectively. In addition, the fluorescent intensity of different NPs in the brain tissue surpassed that in all other dissected organs including the heart, liver, spleen, lung and kidney. The different NPs were also observed to possess certain extent retention in lungs (Fig. 2F–J). This result was in accordance with biodistribution study findings (Fig. S15B†). The results indicate that PEGylated nanoparticles can present a prolonged blood circulation, reduce capture by RES and increase the affinity of targeting ligands with their receptors expressed in the BBB and neutrophils in ischemic brain, which contributes to the significant increasing uptake of the dual-targeting nanoformulation in the brain.

### 3.6. The brain uptake efficacy of 6-coumarin loaded different NPs

To determine the capability of *in vivo* penetration across BBB into the brain, the brain uptake capability of coumarin-6-loaded dual-targeting nanoformulation was further studied by laser scanning confocal microscope (LSM) fluorescent localization of brain slices. Unmodified PEG-PAMAM NPs and single-ligand modified nanocarriers were used as control. Following an intravenous administration, less green fluorescent particles of coumarin-6-loaded PEG-PAMAM NPs was observed in the brain slice (Fig. 3A). As shown in Fig. 3B, Angiopep-2-PEG-PAMAM-NPs showed stronger fluorescence than PEG-PAMAM NPs in the brain slice. This result suggested that Angiopep-2-PEG-PAMAM NPs successfully penetrated into the brain tissue through the endothelial cells of BBB, which proved that the most likely transport mechanism was low density lipoprotein receptor mediated transcytosis process. The fluorescence accumulation coumarin-6-loaded PGP-PEG-PAMAM NPs was higher than that of Angiopep-2-PEG-PAMAM NPs, indicating better brain targeting effect mediated by chemokine receptor relative peptide with specific affinity to neutrophils in ischemic stroke (Fig. 3C, *p* < 0.05). Especially, Angiopep-2-PGP-PEG-PAMAM NPs exhibited markedly stronger fluorescence than that of Angiopep-2-PEG-PAMAM NPs and PGP-PEG-PAMAM NPs (Fig. 3D, *p* < 0.01). As observed in Fig. 3E, the relative fluorescence intensity (*F*/%) of coumarin-6-loaded PEG-PAMAM NPs, coumarin-6-loaded Angiopep-2-PEG-PAMAM NPs, coumarin-6-loaded PGP-PEG-PAMAM NPs and coumarin-6-loaded Angiopep-2-PGP-PEG-PAMAM NPs were 4.73 ± 1.16, 13.62 ± 2.31, 16.46 ± 2.83 and 47.38 ± 3.49, respectively. The significantly enhanced accumulation of Angiopep-2-PGP-PEG-PAMAM NPs in brain indicated that the progressive brain targeting drug delivery system mediated by dual-ligand can be an effective strategy for the treatment of ischemic stroke.

### 3.7. *In vitro* cellular uptake study in BCECs BBB model

**Fig. 4:**
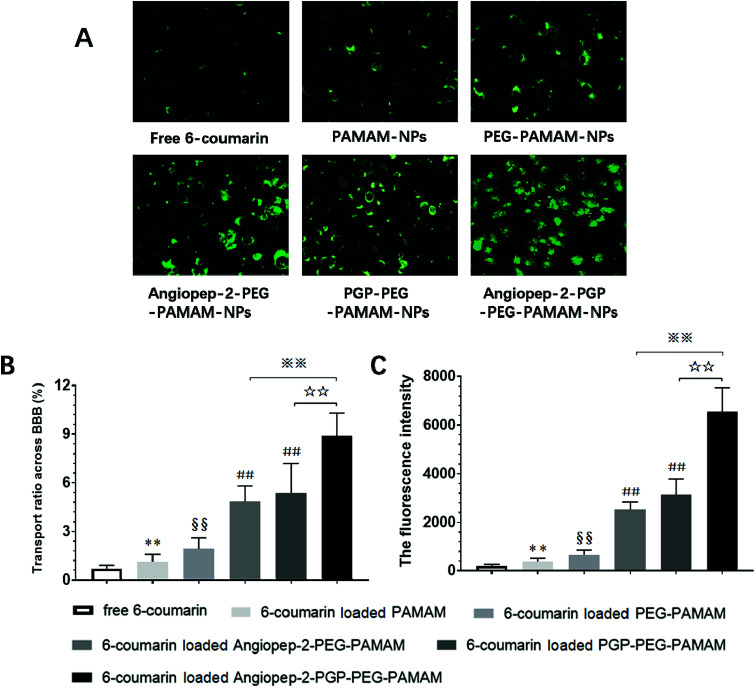
Dual-ligand functionalized nanoformulation transport across the BBB monolayer examined by fluorescent microscopy. (A) Representative fluorescent images of cellular uptake of 6-coumarin-loaded different nanoformulations on BCECs, (B) transport ratios across the BBB monolayer of different 6-coumarin-loaded different NPs and (C) the cumulative fluorescence intensity of cellular uptake of 6-coumarin-loaded different NPs at 4 h. Statistically significant differences by Student's *t*-test when compared to the corresponding value of the control. Data are expressed as means ± SD (standard deviation) (*n* = 5). *p < 0.05, **p < 0.01 *vs.* free 6-coumarin; §§*p* < 0.01 *vs.* 6-coumarin loaded PAMAM NPs; #*p* < 0.05, ##*p* < 0.01 *vs.* 6-coumarin loaded PEG-PAMAM NPs; ☆☆*p* < 0.01 *vs.* 6-coumarin loaded PGP-PEG-PAMAM NPs; ※※*p* < 0.01 *vs.* 6-coumarin loaded Angiopep-2-PEG-PAMAM NPs.

As shown in Fig. 4A and B, the fluorescence intensity study indicated that the cellular uptake of the dual ligands functionalized 6-coumarin loaded nanoformulation was >12 times higher than that of unmodified PEG-PAMAM NPs and nearly 3-fold higher than that of single-ligand modified nanoformulations, respectively. In addition, the cumulative transport amount of the dual-targeting nanoformulation was much higher than the unmodified NPs and single-ligand modified NPs (Fig. 4C). The transport ratio of difference NPs across BBB at 4 h showed 0.69% for free 6-coumarin, 1.12% for PAMAM NPs, 1.93% for PEG-PAMAM NPs, 4.84% for Angiopep-2-PEG-PAMAM NPs, 5.37% for PGP-PEG-PAMAM NPs and 8.89% for Angiopep-2-PGP-PEG-PAMAM NPs (Fig. 4C). The results of transport study demonstrated that the BBB-penetrating ability of Angiopep-2-PGP-PEG-PAMAM NPs was remarkably enhanced owning to dual-ligand mediated targeting mechanism.

### 3.8. Angiopep-2-PGP-STA-PEG-PAMAM NPs salvage brain tissue from ischemic stroke

The sustained drug release profile, low cytotoxicity, favorable pharmacokinetics, and high brain targeting efficiency of Angiopep-2-PGP-STA-PEG-PAMAM NPs make it a promising candidate for further pre-clinical evaluation (*n* = 6 per group). Angiopep-2-STA-PEG-PAMAM NPs, PGP-STA-PEG-PAMAM NPs and Angiopep-2-PGP-STA-PEG-PAMAM NPs were administrated intravenously every day at a dose equivalent to 20 mg kg^−1^ of STA for a total of 3 days, respectively. Free STA (20 mg kg^−1^) and blank Angiopep-2-PGP-PEG-PAMAM NPs (vehicle group) were used as controls in the pharmacodynamics evaluation. The infarct volume, neurological deficit scores and histopathological study in response to cerebral ischemic injury were measured at 24 h after MCAO.

**Fig. 5:**
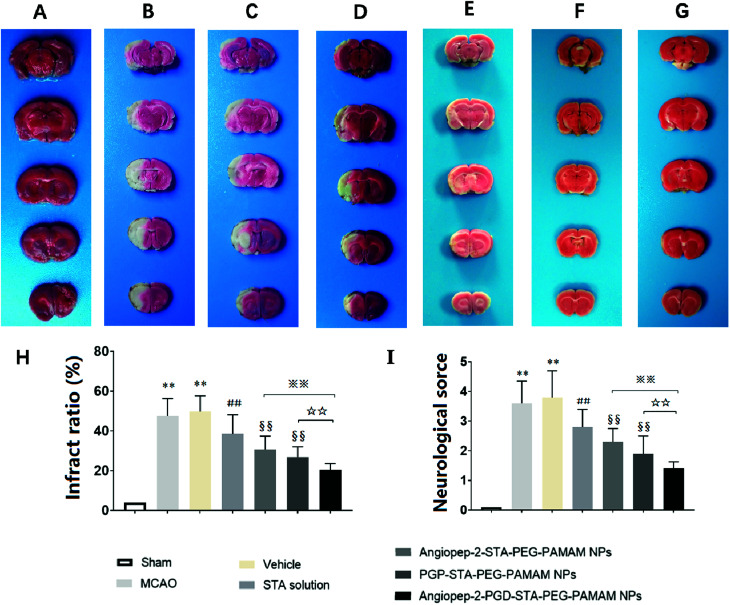
Effect of Angiopep-2-PGP-STA-PEG-PAMAM NPs on infarct ratio (%) and neurological deficits in rats after ischemic stroke. Ischemic brain damage was assessed by 2,3,5-triphenyltetrazolium chloride (TTC) staining on 1 mm brain slices in MCAO rats. The infarct ratio (%) and neurological deficits were significantly reduced by dual-targeting nanoformulation after ischemic stroke (6 animals in each group). Representative TTC-stained brain slices: sham-operated group (A), MCAO group (B), vehicle group (C), free drug group (D), Angiopep-2-STA-PEG-PAMAM NPs group (E), PGP-STA-PEG-PAMAM NPs (F), dual-targeting STA-encapsulated nanoformulation group (G); (H) infarct ratio (%) and (I) neurological deficits at 24 h after MCAO. Statistically significant differences by Student's *t*-test when compared to the corresponding value of the control. Data are expressed as means ± SD (standard deviation) (*n* = 6). **p < 0.01 *vs.* sham-operated group; ##*p* < 0.01 *vs.* MCAO model group; §§*p* < 0.01 *vs.* free STA; ※※*p* < 0.01 *vs.* Angiopep-2-STA-PEG-PAMAM NPs; ☆☆*p* < 0.01 *vs.* PGP-STA-PEG-PAMAM NPs.

As expected, we observed no brain infarction or neurological deficit in the sham-operated group (Fig. 5A), while MCAO surgery induced severe cerebral infarction and neurological deficiency (Fig. 5B and C). There was no significant difference in infarct size and neurological behaviors between MCAO group and the vehicle group (Fig. 5B and C). In contrast, free STA treatment effectively diminished the ischemia-induced infarct volume, neurological deficit score compared with MCAO group and vehicle group (Fig. 5D, *p* < 0.01). Remarkably, two single-ligand modified nanocarriers including Angiopep-2-STA-PEG-PAMAM NPs and PGP-STA-PEG-PAMAM NPs demonstrated near 37% and 45% reduction in infarct ratio and 38% and 49% reduction in neurological scores, respectively, whereas free STA decreased only 21% infarct ratio and 24% neurological scores (Fig. 5E and F, *p* < 0.01). Specifically, dual-targeting nanoformulation exerted the optimum effect in suppressing infarct ratio (58% reduction) and neurological scores (62% reduction) compared with single-ligand modified STA-encapsulated nanocarriers (Fig. 5G, *p* < 0.01).

### 3.9. Effect of Angiopep-2-PGP-STA-PEG-PAMAM NPs on histopathological changes in ischemic stroke

**Fig. 6:**
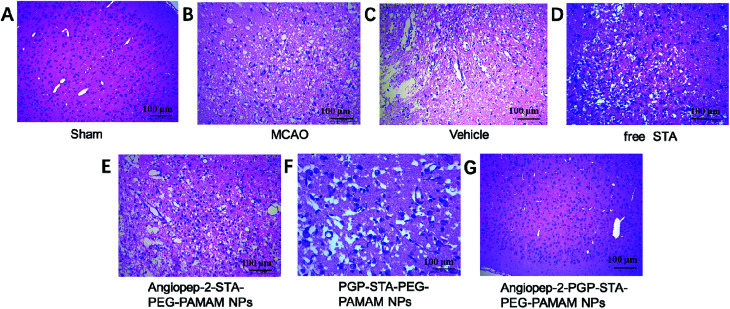
Effect of Angiopep-2-PGP-STA-PEG-PAMAM NPs on histopathological changes in brain tissue in rats after ischemic stroke. HE staining (200×) brain sections and at 24 h after MCAO.

To further investigate the neuroprotective effects of the dual-targeting STA-encapsulated nanoformulation on the rat MCAO model, the morphological changes were observed by H&E staining of the ipsilateral brain 24 h after reperfusion. Blank dual-targeting NPs (vehicle group), free STA and single-ligand modified STA-encapsulated nanocarriers were used as control in histopathological evaluations. Hematoxylin–eosin (H&E) staining of brain tissues suggest similar trends in therapeutic efficacy. Healthy neurons are predominantly observed in brains collected from the sham group and in individual neurons the nucleus was full, nucleolus was clear, and cell outlines were clear (Fig. 6A). While MCAO surgery induced significant neuronal necrosis in the ischemic core and the penumbra, displayed as marked eosinophilic neurons, neuropil damage in cerebral cortex, vacuolation, inflammatory cell infiltration, glial cell proliferation and pyknotic nuclei in H&E stained slides (Fig. 6B and C). In contrast, free STA (Fig. 6D) ameliorated pathological cell death compared with MCAO groups without treatment (Fig. 6B) or treated with blank NPs (Fig. 6C). Nevertheless, Angiopep-2-STA-PEG-PAMAM NPs obviously protected neurons against neuronal damage or neuronal loss compared with equivalent dose of free STA in ischemic stroke induced by surgical MCAO (Fig. 6E). Notably, PGP-STA-PEG-PAMAM NPs had a better therapeutic effect than Angiopep-2-STA-PEG-PAMAM NPs (Fig. 6F). This result strongly suggested that the neutrophils relative receptor-mediated brain-targeting drug delivery to inflammatory sites of ischemic brain as an important mechanism for the protective effects of PGP-STA-PEG-PAMAM NPs on cerebral ischemia-reperfusion injury. Especially, Angiopep-2-PGP-STA-PEG-PAMAM NPs exhibited the strongest inhibition of the number of degenerated neurons and the increase of the number of intact neurons (Fig. 6G), indicating that the anti-ischemic stroke effect of this nanoformulation was markedly elevated by dual-targeting mediated mechanism with Angiopep-2 and PGP.

### 3.10. Dual-targeting STA-encapsulated nanoformulation suppresses inflammatory cytokine levels and neutrophils infiltration in ischemic stroke

**Fig. 7:**
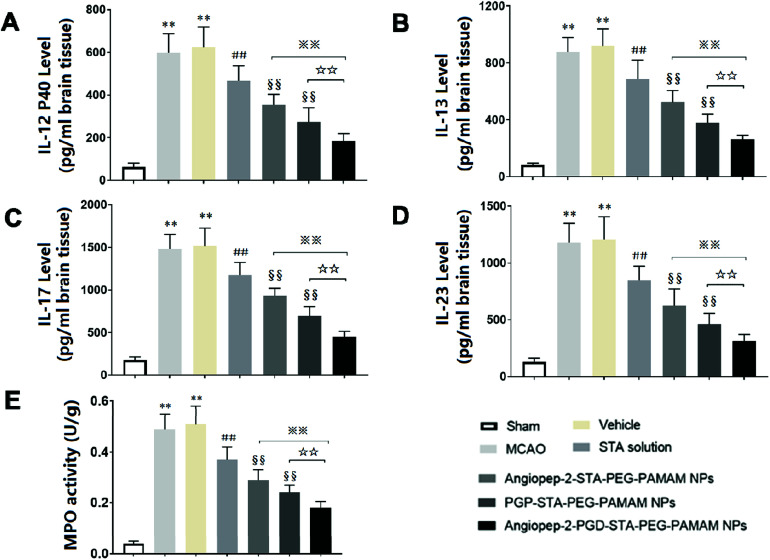
Effect of Angiopep-2-PGP-STA-PEG-PAMAM NPs on inflammatory cytokines levels, including (A) IL-12P40, (B) IL-13, (C) IL-17, (D) IL-23 and (E) MPO activity in brain tissue in rats after ischemic stroke. Statistically significant differences by Student's *t*-test when compared to the corresponding value of the control. Data are expressed as means ± SD (standard deviation) (*n* = 5). **p < 0.01 *vs.* sham-operated group; ##*p* < 0.01 *vs.* MCAO model group; §§*p* < 0.01 *vs.* free STA; ※※*p* < 0.01 *vs.* Angiopep-2-STA-PEG-PAMAM NPs; ☆☆*p* < 0.01 *vs.* PGP-STA-PEG-PAMAM NPs.

Inflammatory cytokines have been thought to play a central role in the processes of neuroinflammation.^3,22^ To better elucidate the neuroprotective effect of dual-targeting STA-encapsulated nanoformulation on ischemic stroke, the levels of inflammatory cytokines were assessed. In sham group, the expression of inflammatory cytokines were almost undetectable in the cerebral hemispheres (Fig. 7A–D). Cerebral ischemia leads to rapid up-regulation of pro-inflammatory cytokines IL-12 p40, IL-13, IL-17 and IL-23 (Fig. 7A–D) in brain tissue. The kinetic of these pro-inflammatory cytokines levels induced by MCAO are in accordance with infarct volume, neurological deficits and histopathological changes. Significant differences were not observed in inflammatory cytokine levels between the MCAO group and vehicle group, indicating that the nanostructure carrier itself does not cause inflammatory alterations. Our findings showed that free STA can ameliorate the neuroinflammation as manifested by the decreased levels of pro-inflammatory cytokines compared with MCAO group (Fig. 7A–D, *p* < 0.01). In comparison to free STA, single-ligand modified STA-encapsulated nanocarriers significantly suppressed pro-inflammatory cytokines in ischemic stroke (*p* < 0.01). Especially, the inhibitory effect of Angiopep-2-PGP-STA-PEG-PAMAM NPs on pro-inflammatory cytokines was prior to that of any single-ligand modified STA-encapsulated nanocarriers (Fig. 7A–D, *p* < 0.01).

The up-regulation of inflammatory cytokines are supposed to have crucial roles in neutrophil infiltration and microglia activation evoked by cerebral ischemia, which can exacerbate ischemic reperfusion injury.^25,48^ MPO activity was used as an indicator of neutrophils infiltration in cerebral ischemia.^3^ PGP, an endogenous tripeptide that acts as a ligand with high affinity to neutrophils in cerebral ischemia. So PGP can be a promising neutrophil anchoring peptide for brain-targeting delivery.^49^ We determined the MPO activity in order to validate and compare the brain-targeting efficiency of single PGP modified STA-encapsulated nanocarriers with synergistic dual-targeting STA-encapsulated nanoformulation, and further evaluate their inhibitory effects on neutrophil infiltration in ischemic stroke. We found that MPO level as an indicator of neutrophils infiltration in MCAO group and vehicle group were dramatically higher than that of sham group (Fig. 7E, *p* < 0.01). In contrast, administration of free STA obviously reduced MPO activity compared with MCAO group and blank vehicle group. Specially, treatment with Angiopep-2-STA-PEG-PAMAM NPs and PGP-STA-PEG-PAMAM NPs remarkably down-regulated MPO activity compared with free STA due to active brain targeting drug delivery (Fig. 7E, *p* < 0.01), whereas PGP-STA-PEG-PAMAM NPs displayed much lower MPO activity than that of Angiopep-2-STA-PEG-PAMAM NPs in the brain tissue, owing to the high affinity of PGP peptide with the receptors expressing on the surface of neutrophil. In comparison to single-ligand modified STA-encapsulated nanocarriers, dual-targeting STA-encapsulated nanoformulation was the most effective in suppressing MPO activity, which exhibited an excellent co-mediated brain-targeting effect. These results were consistent with those of histological studies.

Here, we demonstrated that Angiopep-2 and PGP co-modified nanoformulation can successfully delivery STA to the inflammatory sites of the ischemic brain. These results suggested that another crucial mechanism underlying the protective effect of dual-ligand modified STA-encapsulated nano delivery system on ischemic stroke involved in the remarkable down-regulation of inflammatory cytokines and neutrophils infiltration.

### 3.11. Angiopep-2-PGP-STA-PEG-PAMAM NPs inhibits intracellular calcium overload

**Fig. 8:**
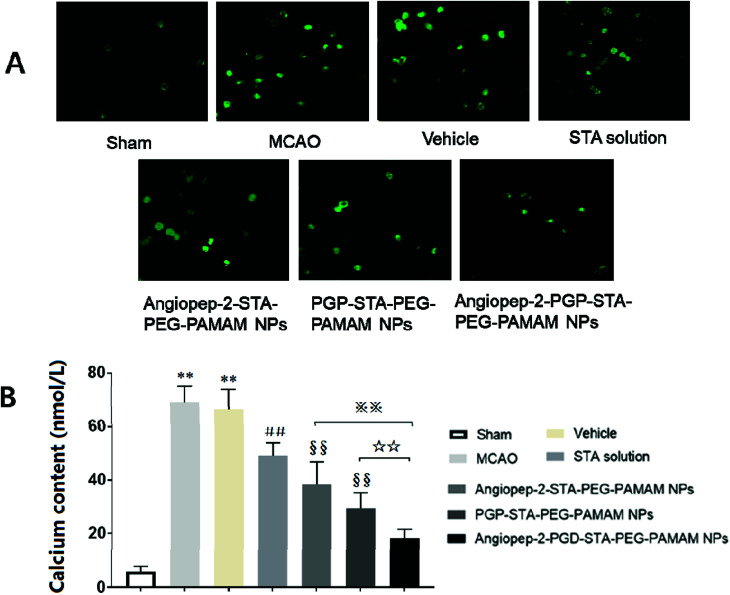
Effect of the dual-targeting STA-encapsulated nanoformulation on intracellular Ca^2+^ level in hippocampal neurons (A and B). The fluorescence intensity of [Ca^2+^]i in hippocampal neurons was determined by laser scanning confocal microscopy. High calcium content was obviously observed at 24 h after OGD/RP, MPO level was also markedly increased at 24 h after MCAO, which were significantly downregulated by the dual-targeting STA-encapsulated nanoformulation. Statistically significant differences by Student's *t*-test when compared to the corresponding value of the control. Data are expressed as means ± SD (standard deviation) (*n* = 5). **p < 0.01 *vs.* Normal group; ##*p* < 0.01 *vs.* MCAO group; §§*p* < 0.01 *vs.* free STA; ※※*p* < 0.01 *vs.* Angiopep-2-STA-PEG-PAMAM NPs; ☆☆*p* < 0.01 *vs.* PGP-STA-PEG-PAMAM NPs.

Calcium is an important second messenger involved in neurotransmitter release and signal transduction. Numerous studies have proved that the alteration of intracellular Ca^2+^ homeostasis plays a central role in initiating the apoptotic response. Elevation of [Ca^2+^]i leads to destabilization of the neuronal cell structure and cause cell damage, eventually cell death.^50^ To further explore the neuroprotection mechanisms of the dual-targeting STA-encapsulated nanoformulation on the intracellular [Ca^2+^]i overload, oxygen-glucose deprivation/reperfusion (OGD/RP) injury model was performed. Our results showed that [Ca^2+^]i level in hippocampal neurons was markedly up-regulated after OGD/RP (Fig. 8A and B, *p* < 0.01). In contrast, the [Ca^2+^]i overload induced by OGD/RP was attenuated after treatment with free STA (Fig. 8A and B, *p* < 0.01). Moreover, single-ligand modified STA-encapsulated nanocarriers significantly depressed [Ca^2+^]i elevation compared with free STA (Fig. 8A and B, *p* < 0.01). Remarkably, maximum inhibition effect was observed after administration of the dual-targeting STA-encapsulated nanoformulation at OGD 2 h/RP 24 h (Fig. 8A and B, *p* < 0.01). Hence, we inferred that the one of the important neuroprotection mechanism of Angiopep-2-PGP-STA-PEG-PAMAM NPs on cultured rat hippocampal neurons injured by OGD/RP may be related to inhibition of intracellular calcium overload.

### 3.12. Effect of Angiopep-2-PGP-STA-PEG-PAMAM NPs on TLR2, TLR4, TLR5, HMGB1, MyD88 and TRIF expressions

It is generally evidenced that inflammatory responses and cell apoptosis in neuronal injury after ischemia/reperfusion are mediated by HMGB1, Toll-like receptors and NF-κB activation.^23,26^ Recent researchs considered that TLR2/4 signaling could activate both the MyD88-dependent pathway and the TRAM/TRIF-dependent signaling pathway in macrophages.^27^ TRAF6 is required for MyD88-dependent TLRs/NF-κB activation.^30^ The activation of NF-κB induces the up-regulation of leukocyte adhesion molecules and the production of pro-inflammatory cytokines in neuronal injury after ischemia/reperfusion, thereby, promoting inflammation.^28^ The activation of these signaling pathways could be induced by oxidative stress, neuronal apoptosis, inflammatory cytokines and increases of intracellular Ca^2+^ levels.^28,29^ Given the fact that calcium overload usually results from alterations of intracellular signaling pathways, we further hypothesize that the dual-targeting STA-encapsulated nanoformulation can alter signaling pathways involved in inflammatory responses, neutrophils infiltration and calcium overload, for example, the well-established HMGB1/Toll-like receptors/NF-κB activation pathway.^23,26,51^

**Fig. 9:**
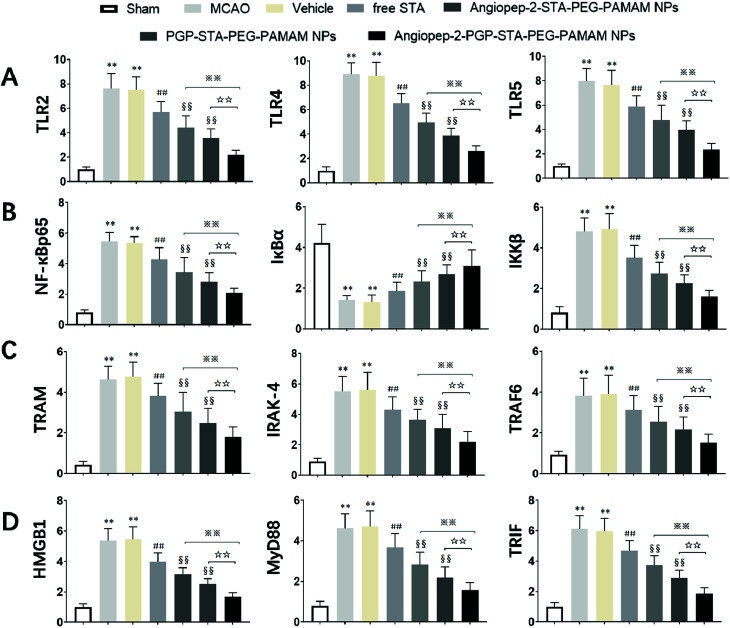
Effect of Angiopep-2-PGP-STA-PEG-PAMAM NPs on mRNA expression of (A) TLR2, TLR4 and TLR5; (B) NF-κBp65, IκBα and IKKβ; (C) TRAM, IRAK-4 and TRIF6; (D) HMGB1, MyD88 and TRIF in brain tissue at 24 h after cerebral ischemia in rats. The mRNA levels in cerebral homogenates were determined using real-time PCR following normalization to housekeeping gene. Statistically significant differences by Student's *t*-test when compared to the corresponding value of the control. Data are expressed as means ± SD (standard deviation) (*n* = 5). **p < 0.01 *vs.* sham group; ##*p* < 0.01 *vs.* MCAO group; §§*p* < 0.01 *vs.* free STA; ※※*p* < 0.01 *vs.* Angiopep-2-STA-PEG-PAMAM NPs; ☆☆*p* < 0.01 *vs.* PGP-STA-PEG-PAMAM NPs.

**Fig. 10:**
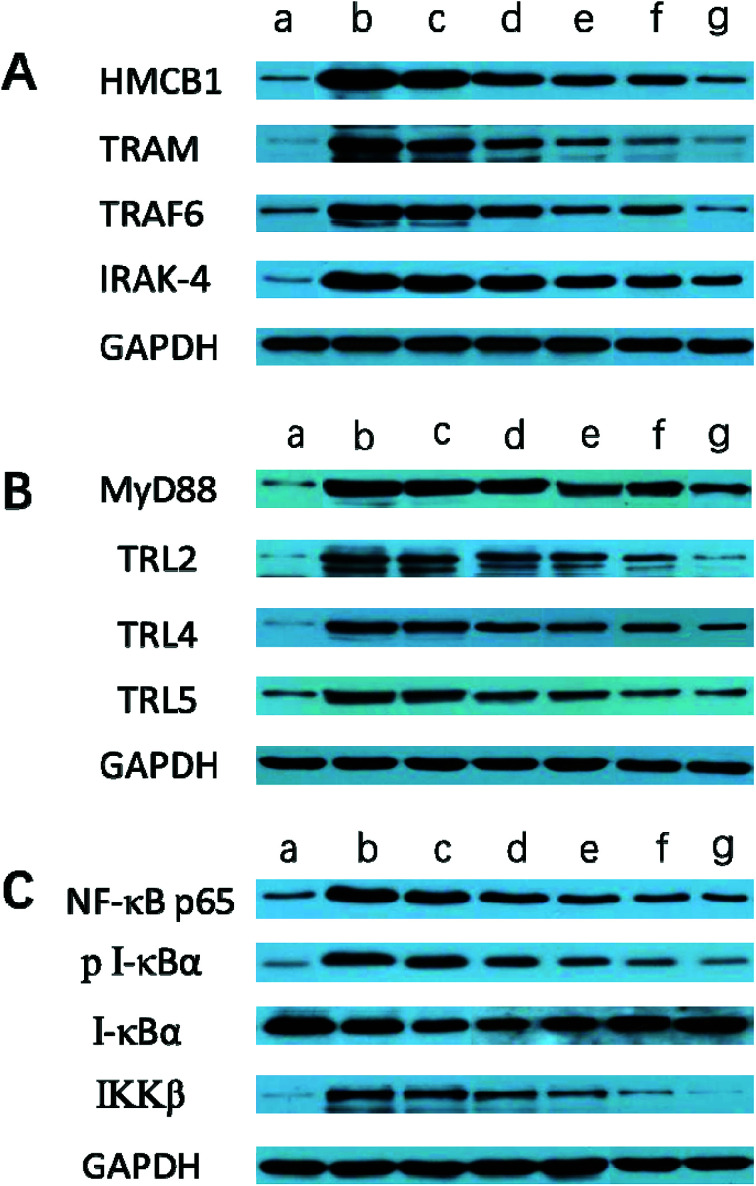
Effect of Angiopep-2-PGP-STA-PEG-PAMAM NPs on the protein expressions of (A) HMGB1, TRAM, TRIF6 and IRAK-4, (B) MyD88, TLR2, TLR4 and TLR5, (C) NF-κBp65, *p*-IκBα, IκBα and IKKβ in brain tissue at 24 h after ischemic stroke. The protein expressions of HMGB1, TRAM, TRAF6, IRAK-4, MyD88, TLR2, TLR4, TLR5, NF-κBp65, *p*-IκBα, IκBα and IKKβ were examined by western blotting ((A–C) (a) sham group; (b) MCAO group; (c) vehicle group; (d) free STA; (e) Angiopep-2-STA-PEG-PAMAM NPs; (f) PGP-STA-PEG-PAMAM NPs; (g) Angiopep-2-PGP-STA-PEG-PAMAM NPs).

With this in mind, we investigated whether treatment with Angiopep-2-PGP-STA-PEG-PAMAM NPs would alter the endogenous mRNAs and proteins expression of involved in the HMGB1/TLRs/MyD88/TRIF/NF-κB signaling pathway after ischemic stroke. In this study, we evaluated the mRNA and protein expressions of major subgroups of HMGB1/TLRs/MyD88/TRIF signaling pathways involved in inflammation and cell apoptosis after ischemic stroke. As observed in Fig. 9A and D, we found that the mRNA expressions of HMGB1, TLR2, TLR4, TLR5, MyD88 and TRIF were remarkably up-regulated after MCAO at 24 h compared with sham group (*p* < 0.05). The results of real-time PCR were consistent with protein expression levels as measured with western blot (Fig. 10A and B). The increased mRNA and protein expressions of the HMGB1/TLRs/MyD88/TRIF/NF-κB signaling pathways could contribute to brain injury through their production such as oxidative stress, inflammatory cytokines and increases of intracellular Ca^2+^ levels.^25,50^ There was no significant difference found between the MCAO group and the vehicle group (Fig. 9A, D, 10A and B). By contrast, free STA effectively inhibited the mRNA and protein expressions of TLR2, TLR4, TLR5, HMGB1, MyD88 and TRIF compared with the MCAO group (Fig. 9A and D, *p* < 0.01). Interestingly, treatment with single-ligand modified STA-loaded nanocarriers notably suppressed the mRNA and protein expressions of TLR2, TLR4, TLR5, HMGB1, MyD88 and TRIF compared with free STA (Fig. 9A, D, 10A and B, *p* < 0.01). Especially, the inhibitory effect of PGP-STA-PEG-PAMAM NPs on the mRNA and protein expressions of HMGB1, TLR2, TLR4, TLR5, MyD88 and TRIF was much better than that of Angiopep-2-STA-PEG-PAMAM NPs, suggesting PGP as a neutrophil specific targeting ligand can further delivery drug to inflammatory sites of ischemic brain. In particular, Angiopep-2-PGP-STA-PEG-PAMAM NPs was the most effective in reducing the mRNA and protein expressions of TLR2, TLR4, TLR5, HMGB1, MyD88 and TRIF validating the synergistic dual-targeting effect (Fig. 9A, D, 10A and B, *p* < 0.01).

### 3.13. Effect of Angiopep-2-PGP-STA-PEG-PAMAM NPs on TRAM, IRAK-4, TRAF6, NF-κBp65, IκBα and IKKβ expressions

We also investigated whether Angiopep-2-PGP-STA-PEG-PAMAM NPs would alter endogenous expression of TRAM, IRAK-4, TRAF6, NF-κBp65, IκBα and IKKβ in the ischemic hemisphere. As observed in Fig. 9B, C, 10B and C, cerebral ischemia/reperfusion induced an abrupt increase of mRNA and protein expressions of TRAM, IRAK-4, TRAF6, NF-κBp65 and IKKβ compared to the sham group, which can be down-regulated by free STA (*p* < 0.01). Specially, single-ligand modified STA-encapsulated nanocarriers remarkably suppressed the mRNA and protein expressions of TRAM, IRAK-4, TRAF6, NF-κBp65 and IKKβ compared with free STA (Fig. 9B and C, *p* < 0.01). Notably, synergistic dual-targeting STA-encapsulated nanoformulation displayed the strongest inhibition of the mRNA and protein levels of TRAM, IRAK-4, TRAF6, NF-κBp65 and IKKβ (Fig. 9B and C, *p* < 0.01). These results were consistent with that of western blot analysis (Fig. 10B and C).

On the other hand, the degradation and phosphorylation of IκBα were distinctively induced in ischemia/reperfusion rats compared with sham group (Fig. 9B and C, *p* < 0.01). By contrast, real-time PCR of IκBα (Fig. 9B) and western blot of phospho-IκBα (Fig. 10C) indicated that administration of free STA obviously reduced the phosphorylation and degradation of IκBα (*p* < 0.01). Notably, single-ligand modified STA-encapsulated nanocarriers significantly up-regulated the level of IκBα and inhibited the overexpression of phospho-IκBα compared with free STA (*p* < 0.01). In particular, Angiopep-2-PGP-STA-PEG-PAMAM NPs was the most effective in up-regulating IκBα level and decreasing the overexpression of phospho-IκBα. Our finding indicated that Angiopep-2-PGP-STA-PEG-PAMAM NPs markedly suppressed NF-κB signaling pathway in ischemic stroke by inhibiting its binding to target DNA and suppressing IκBα phosphorylation/degradation, IKKβ activity and relative inflammatory mediators in ischemic hemisphere. The above results implied that Angiopep-2-PGP-STA-PEG-PAMAM NPs might exert its inhibitory effect on inflammatory mediators and cell apoptosis through blockade of HMGB1/TLRs/MyD88/TRIF/NF-κB signaling pathways.

## 4. Conclusion

In summary, we successfully developed a low density lipoprotein receptor and neutrophils receptor co-mediated brain-targeting drug-loaded nanoformulation (Angiopep-2-PGP-STA-PEG-PAMAM NPs). The dual-targeting nanoformulation presented notable advantages: (i) improved bioavailability (relative bioavailability of 318%); (ii) impressively high drug loading efficiency (nearly 60% of free drug, w/w); (iii) facilitating the penetration of pharmaceutical molecules through the BBB (12-fold higher brain fluorescent accumulation than PAMAM NPs and 3-fold higher brain fluorescent accumulation than single-ligand modified nanocarriers); (iv) significantly reducing the cytotoxicity of PAMAM dendrimer (over 70% cell viability); (v) avoiding recognition and uptake by the reticuloendothelial system, thereby remarkably prolonging the circulation time (MRT extended 6.7 times) of drugs in the blood and increasing half-life (extended 5.3 times). To the best of our knowledge, the present study is the first in depth investigation from cell and molecular level of how low density lipoprotein receptor and neutrophils receptor co-mediated transportation of low bioavailability small-molecule drug brain-targeting nanocarrier correlate with their *in vivo* anti-ischemic stroke efficacy and protective mechanism on inflammatory response in ischemic stroke. This dual-ligand modified nanoformulation with impressively high drug loading capacity and efficiently brain drug delivery to ischemic stroke area can provide excellent anti-stroke activity and suppress the inflammatory cytokines levels, neutrophils infiltration and calcium overload in ischemic brain tissue. The results indicated that the potent anti-inflammatory effects of the dual-targeting nanoformulation against ischemic stroke might attribute to its blockade of HMGB1/TLRs/MyD88/TRIF/NF-κB signaling pathways. Although these effects most probably contributed to its therapy of ischemic stroke, there are other potential mechanisms in which Angiopep-2-PGP-STA-PEG-PAMAM NPs that could have been involved. Therefore, our future studies will investigate other signaling pathways involved in inflammation cascade and brain injury in the pathological progress of CNS diseases. The current findings may encourage further researches into the application of co-mediated dual-targeting nanoformulation for simultaneous delivery of therapeutic agents for noninvasive and precise therapy of CNS diseases.

## Author contributions

Yanxin Dang, Yutao Li, and Dandan Han carried on most of the research. Xin Liu composed the paper. Chiying An, Yuan Xu and Haijing Zhong completed the pharmacology experiments. Mewand Khan Karim Khan assisted in the characterization for the polymers. W.C. assisted in the pharmacokinetics and biodistribution. Fengming Zhang and Fengjuan Zou synthesized the mentioned polymers. Xin Liu and Xiaojun Sun supervised the research work.

## Conflicts of interest

The authors declare that they have no conflict of interest.

## Supplementary Material

RA-009-C8RA06688D-s001
