# Supplementary material for: Neutrophil-mediated and low density lipoprotein receptor-mediated dual-targeting nanoformulation enhances brain accumulation of scutellarin and exerts neuroprotective effects against ischemic stroke
Source: RSC Adv. 2019 Jan 10;9(3):1299–318. doi: 10.1039/c8ra06688d (PMC9059646; doi:10.1039/c8ra06688d)
Supplement: RA-009-C8RA06688D-s001 [file RA-009-C8RA06688D-s001.pdf]

Electronic Supplementary Material (ESI) for RSC Advances.
This journal is © The Royal Society of Chemistry 2018

Supplementary Information

1. Abbreviations

STA, scutellarin; BBB, blood-brain barrier; PEG, polyethylene glycol; PAMAM, poly amidoamine; LRP, low density lipoprotein receptor-mediated peptide; PGP, N-acetylated proline-glycine-proline; Angiopep-2-PGP-PEG-PAMAM NPs, Angiopep-2 and PGP co-modified PEG-PAMAM nanoformulation; MCAO, Middle cerebral artery occlusion; IL, interleukin; IRAK, interleukin-1 receptor associated kinase; TRAF, TNF receptor-associated factor; TTC, 2, 3, 5-triphenyltetrazolium chloride; TRAM, Toll-like receptor adaptor molecule; TRIF, Toll receptor associated activator of interferon; NF-*κ*Bp65, nuclear factor-kappaBp65; I*κ*B, inhibitor of *κ*B; IKKβ, I*κ*B kinase β; TUNEL, terminal deoxynucleotidyl transferase (TdT)-mediated dUTP-biotin nick end labeling; DAB, 3, 3′-diaminobenzidine; HMGB1, High mobility group box 1; TLRs/MyD88/NF-κB, Toll-like receptors-dependent MyD88 induced NF-κB

2 Materials and Methods

2.1 Synthesis and Characterization of Hydroxyl-terminated G 5.0 PAMAM Dendrimer

Hydroxyl-terminated G 5.0 PAMAM dendrimer was synthesized according to the situ branch cell method mentioned in the previous reports [1, 2]. The reaction is a two-step iterative process for constructing PAMAM dendrimers possessing either terminal ester or amine groups. This method involves (1) alkylation with methyl acrylate (MA), and (2) amidation with ethylenediamine (EDA). The reactants were repeatedly washed with methanol (once every 2 h) and evaporated under reduced pressure at 40℃ for 8 h to remove the methanol solvent and excess MA. The supernatant was washed with methanol and evaporated under reduced pressure to remove ether and methanol to obtain a higher purity of hydroxyl-terminated G 0.5 PAMAM. The product of hydroxyl-terminated G 0.5 PAMAM was obtained with the yield of 98.7%.

The ^1H NMR and ^{13}C NMR spectra of hydroxyl-terminated G5.0 PAMAM dendrimer (dissolved in D_2O) were analyzed in Bruker AVANCE 500 MHz NMR spectrometer (Switzerland). The sample of hydroxyl-terminated G5.0 PAMAM was ground, mixed with KBr and pressed into pellets. The infrared spectra were recorded in the spectral range of 400～4000 cm^{-1}, using a Nicolet 5700 FT-IR spectrometer with a resolution of 2 cm^{-1}.

2.2 Synthesis and Characterization of NHS-PEG-MAL-PAMAM Diblock Copolymers

PEGylated PAMAM copolymers were designed and synthesized by conjugating bifunctional NHS-PEG_{3400}-MAL to the amine groups of the synthetic hydroxyl-terminated G5.0 PAMAM dendrimers. Briefly, the synthesized hydroxyl-terminated G 5.0 PAMAM reacted to NHS-PEG-MAL (MW 3400) with the molar ratio of 1: 8 in phosphate buffer (PBS, pH 8.0) under magnetic stirring in the dark for 12 h at room temperature. In this stage, the PAMAM primary amine groups react exclusively to terminal NHS groups of bifunctional PEG. The reaction mixture was transferred into an ultrafiltration tube (Millipore, USA, 50 KDa) and centrifuged at 8000 rpm for 30 min in order to separate the unreacted PEG. The yield of NHS-PEG-MAL-PAMAM was 57.6% (percentage of the total amount of raw materials, PAMAM and NHS-PEG_{3400}-MAL, W/W).

The ^1H NMR and ^{13}C NMR spectra of NHS-PEG-MAL-PAMAM diblock copolymers (dissolved in D_2O) were analyzed in Bruker AVANCE 500 MHz NMR spectrometer (Switzerland). The infrared spectra were recorded in the spectral range of 400～4000 cm^{-1}, using a Nicolet 5700 FT-IR spectrometer with a resolution of 2 cm^{-1}.

2.3 Cytotoxicity assay in vitro

The cytotoxicity of dual-ligand modified nanoformulation and single-ligand modified nanocarriers on brain capillary endothelial cells (BCECs) were evaluated as previously described [3] by MST assay using hydroxyl-terminated G5.0 PAMAM NPs and PEG-PAMAM NPs as control groups. Briefly, BCECs were seeded in 96-well plates with a density of 2×10^3 cells/well in 200 μL DMEM medium and incubated

for 24 h. Then cells were treated with blank vehicles such as G5.0 PAMAM NPs, PEG-PAMAM NPs, Angiopep-2-PEG-PAMAM NPs, PGP-PEG-PAMAM NPs and Angiopep-2-PGP-PEG-PAMAM NPs at a series of concentrations ranging from 0.001 to 1000 μ g/mL in culture medium, respectively. After 48 h incubation, the media were replaced with fresh culture media containing MST solution (0.5 mg/mL). After an additional incubation of cells for 4 h, the medium was removed and DMSO was added. The OD values were measured by a microplate reader (Spectra Max M3, Molecular Devices, USA) at wavelength 570 nm, and cell viability was calculated by dividing the OD values of samples with the OD values of blank.

2.4 Stability and Drug Release

To evaluate the stability of Angiopep-2-PGP-STA-PEG-PAMAM NPs, three PBS solutions (0.1 M, pH 5.0, pH 7.4 or pH 9.0) were prepared. 10 mg Angiopep-2-PGP-STA-PEG-PAMAM NPs were suspended in 15 mL of each PBS solution with continuous stirring under 120 rpm at 37°C. The size and zeta potential of Angiopep-2-PGP-STA-PEG-PAMAM NPs were measured by dynamic light scattering (DLS) on days 0, 1, 2, 3, and 5 to monitor its stability [4].

Cumulative STA release from Angiopep-2-PGP-STA-PEG-PAMAM NPs was determined by dispersing 1 mg/ml of the NPs in 0.01 M phosphate buffer (pH 7.4), and incubating it at 37°C. Aliquots of the dispersion were withdrawn at 0 min, 15 min, 30 min, 1 h, 2 h, 4 h, 8 h, 12 h, 24 h, 48 h and 72 h, and centrifuged at 15,000 rpm for 25 min. Volume of the dispersion withdrawn was replaced with fresh phosphate buffer. The supernatant was collected and STA concentrations were quantitated by HPLC. The experiments were performed in triplicates.

2.5 Pharmacokinetics and biodistribution of dual-targeting STA-encapsulated nanoformulation in cerebral ischemia rats

Pharmacokinetics and biodistribution experiments were performed in cerebral ischemia SD rats (n = 6 each group). Briefly, Free STA (20 mg/kg) suspended in CMC-Na solution (0.5%, w/v) Angiopep-2-STA-PEG-PAMAM NPs, PGP-STA-PEG-PAMAM NPs and Angiopep-2-PGP-STA-PEG-PAMAM NPs (containing STA, 20 mg/kg) dispersed in saline solution were administered to each group through the

tail vein injection at a single dose, respectively. Around 200 μL of blood was collected from the jugular vein into heparinized centrifuge tube at predetermined time points (5, 15, 30, and 45 min; 1, 2, 4, 6, 8, 12, 24 and 36 h). The plasma was prepared for HPLC-MS/MS by removing protein with a precipitation method [5]. The prepared samples were kept in an auto-sampler at 4℃ until injection, at which time 10 μL of supernatant fluid was injected into the HPLC/MS/MS system. The experiments were performed in triplicates.

In tissue distribution study (n = 6 each group), SD rats were sacrificed at predetermined time points, and then the brain tissue and other major organs including heart, liver, spleen, lung, brain and kidney were excised, followed by quick washing with cold saline to remove surface blood and stored at - 20℃ until analysis.

Briefly, blood was cleared prior to organ homogenization. The tissues were homogenized in ice saline, and then extracted three times with acetic ether. The organic phase was combined after centrifugation at 14,000 rpm for 10 min, and dried under a stream of nitrogen at 37℃. The dry residues were dissolved in 100 μL methanol, and the concentration of STA was determined by the UPLC-QTOF-MS for analysis. The experiments were performed in triplicates.

2.6 Induction of focal cerebral ischemia and experimental design

Briefly, the right common carotid artery (CCA), external carotid artery (ECA) and internal carotid artery (ICA) of SD rats were isolated via a ventral midline incision. Branches of ECA were cauterized. The ECA was ligated and cut off at the distance of 5 mm from crotch of CCA. A nylon monofilament (φ 0.22～0.24 mm) with a rounded tip was inserted into right ICA about 18～20 mm through the broken end of ECA, until laser Doppler flow metry as an abrupt 80%～90% reduction of cerebral blood flow, indicating the occlusion of the origin of middle cerebral artery. Sham-operated control rat received the same procedure except filament insertion. After 2 h of ischemia, the nylon suture was withdrawn to establish reperfusion.

For ICR mice, after 1 h of ischemia, the nylon suture was withdrawn to establish reperfusion.

Rats were randomly divided into 7 groups (n = 6 per group): group 1: sham-operated group injected with normal saline in a comparable volume; group 2: model group; group 3: vehicle control group administered with blank Angiopep-2-PGP-PEG-PAMAM NPs dissolved in normal saline; group 4: treated with free STA (20 mg/kg); group 5: treated with Angiopep-2-STA-PEG-PAMAM NPs (every day at a dose equivalent to 20 mg/kg of STA for a total of 3 days); group 6: treated with PGP-STA-PEG-PAMAM NPs (every day at a dose equivalent to 20 mg/kg of STA for a total of 3 days) and group 7: treated with Angiopep-2-PGP-STA-PEG-PAMAM NPs (every day at a dose equivalent to 20 mg/kg of STA for a total of 3 days). All formulations were administered *via* the intravenous route at the time of reperfusion following 2 h of ischemia/reperfusion, respectively.

2.7 Assessment of neurological function and infract volume

The neurological function assessment was carried out by an examiner blinded to the experimental groups at 24 h after MCAO, all of the 70 rats from each group (n = 10) were assessed on a modified scoring system that developed from our previous studies [6, 7], as follows: 0, normal spontaneous movements; 1, difficulty in fully extending the contralateral forelimb; 2, unable to extend the contralateral forelimb; 3, mild circling to the contralateral side; 4, severe circling to the contralateral side; 5, an animal was unresponsive to noxious stimulus or unconsciousness.

Infarct volume (mm^3) was determined by 2, 3, 5-triphenyltetrazolium chloride (TTC) after the neurological assessment (n = 6 per group). Animals were euthanized and the brains quickly collected. Brain tissue was sliced into 1 mm-thick slice, stained with a 2% solution of TTC at 37℃ for 30 min, followed by fixation with 4% paraformaldehyde [7]. Normal tissue was stained deep red, while the infarct area was stained a pale gray color. TTC-stained sections were photographed and infarct volumes (mm^3) were quantified using image analysis software (Image-Pro Plus 5.1, USA) to calculate the infarct ratio (%).

3 Results

3.1 Characterization of G5.0 PAMAM

The successful synthesis of hydroxyl-terminated G5.0 PAMAM polymers was demonstrated by multiple characteristic peaks belonged to the branching units of PAMAM. Detailed spectral analysis is presented in the Figure S2 and Figure S4. In ^{1}H NMR spectrum (Figure S2), the multiple peaks between 2.3 and 3.2 ppm belonged to the methylene protons of branching units of PAMAM. The proton of terminal amino group presented the corresponding peak at 2.327 ppm (Figure S2). The ^{13}C NMR data (Figure S3) shows a single peak of at 174.96/174.51 ppm attributed to C=O group in PAMAM segment. Significant FT-IR spectral changes were detected in the region of 1000～4000 cm^{-1} containing $-NH_2$, -CONH, $-CH_2-$, C-N, tertiary amine and other functional characteristic groups (Figure S4), which consistent with the theoretical structure of G5.0 PAMAM. The peak at 1638.01 cm^{-1} pertained to the amide bond, the peak at 1560.34 cm^{-1} is the stretching vibration of N-H bond and C-H bond in the amide bond; the peak at 1466.52 cm^{-1} corresponded to the bending vibration of $-CH_2$. The peaks at 1198.74 cm^{-1} and 1127.36 cm^{-1} are the stretching vibrations of primary and tertiary amines, respectively.

3.2 Characterization of NHS-PEG-MAL-PAMAM Diblock Copolymers

PEGylated PAMAM nanocarriers were designed and synthesized by conjugating bifunctional NHS-PEG_{3400}-MAL to the amine groups of the synthetic G5.0 PAMAM dendrimers. The chemical structures of PEG-PAMAM were confirmed by ^{1}H NMR, ^{13}C NMR, thin-Layer Chromatography (TLC) and infrared spectrogram (IR) (Figure S5-Figure S8). The successful synthesis of NHS-PEG-MAL-PAMAM diblock copolymers was confirmed by the appearance of a signal at 3.578 ppm (^{1}H NMR) that corresponded to the methylene protons of NHS-PEG-MAL (Figure S5).[28] Furthermore, the repeat units of PEG presented a sharp peak at 3.7 ppm (^{1}H NMR, Figure S5), showing that the MAL group had reacted with the thiol group of Angiopep-2.[29] The methene group ($-CH_2-$) of NHS-PEG-MAL segment appeared at 69.68 ppm (^{13}C NMR, Figure S6). As shown in FT-IR spectrum (Figure S8), NHS-PEG-MAL-PAMAM showed its unique characteristic peaks in the region from 1400 cm^{-1} to 1100 cm^{-1}. Furthermore, stretching peak of primary amines near 1198 cm^{-1} attributed to hydroxyl-terminated G5.0 PAMAM disappeared, and a new peak was

observed at 3429.32 cm^{-1}, which was just the primary amino peak of hydroxyl-terminated G5.0 PAMAM present at 3288.68 cm^{-1} (Figure S8) and red-shifted to 3439.32 cm^{-1} in IR spectrum of PEG-PAMAM (Figure S8). The peak at 2918.13 cm^{-1} was the stretching vibration peak of $-CH_2$ belonged to PEG-PAMAM, and the peak at 1101.92 cm^{-1} is the stretching vibration peak of C-O-C in PEG. So it can be concluded that NHS-PEG_{3400}-MAL reacted with the primary amino groups on the surface of hydroxyl-terminated G5.0 PAMAM.

Supplementary Figures

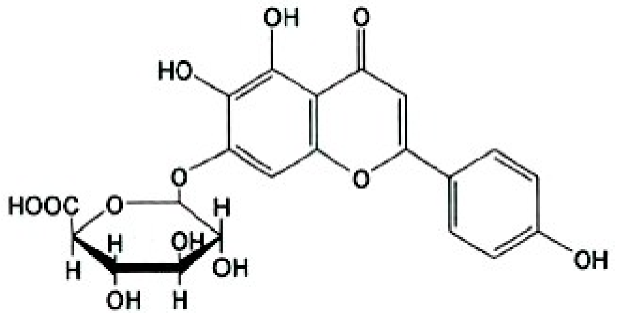

Fig. S1 Chemical structure of scutellarin (STA)

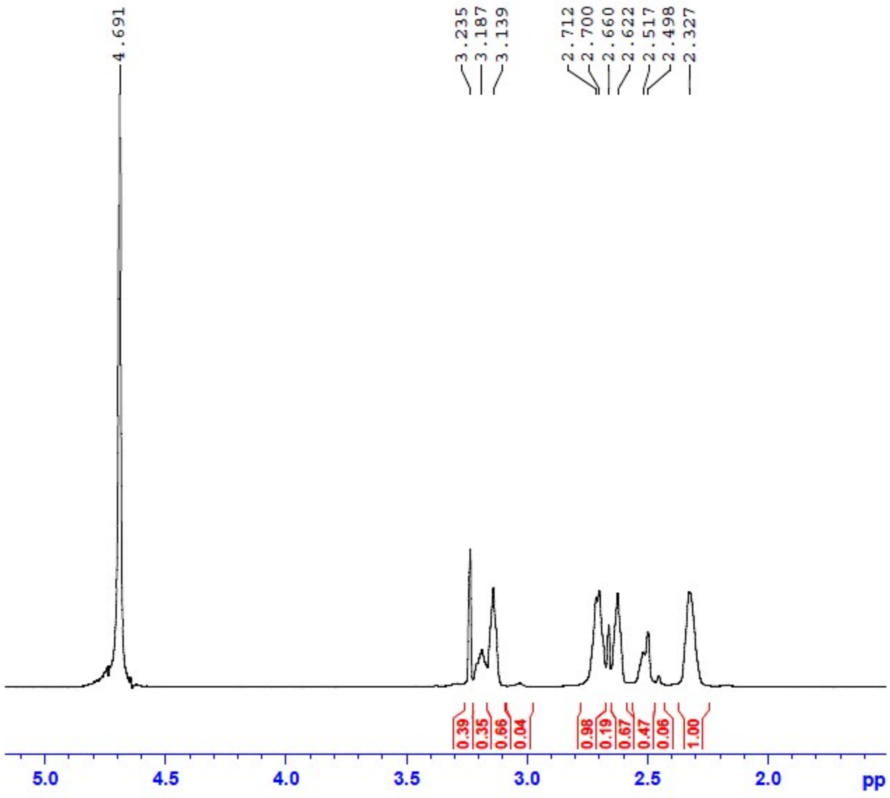

Fig. S2 ^{1}H-NMR spectra of synthesized hydroxyl-terminated G5.0 PAMAM dendrimer

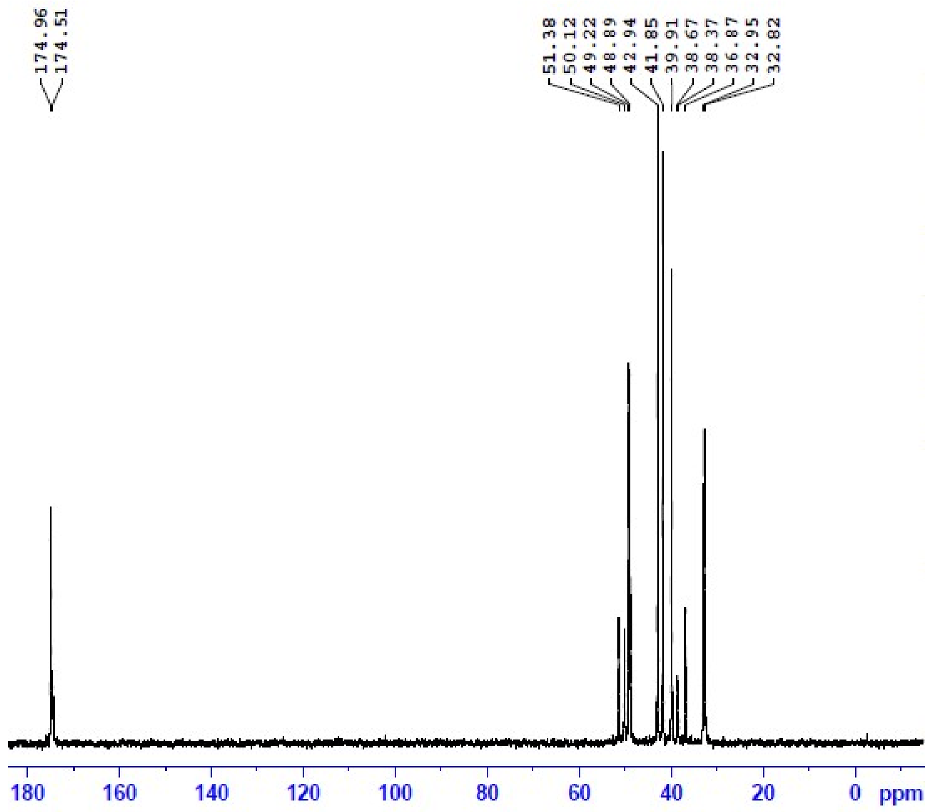

Fig. S3 ^{13}C NMR spectra of synthesized hydroxyl-terminated G5.0 PAMAM dendrimer.

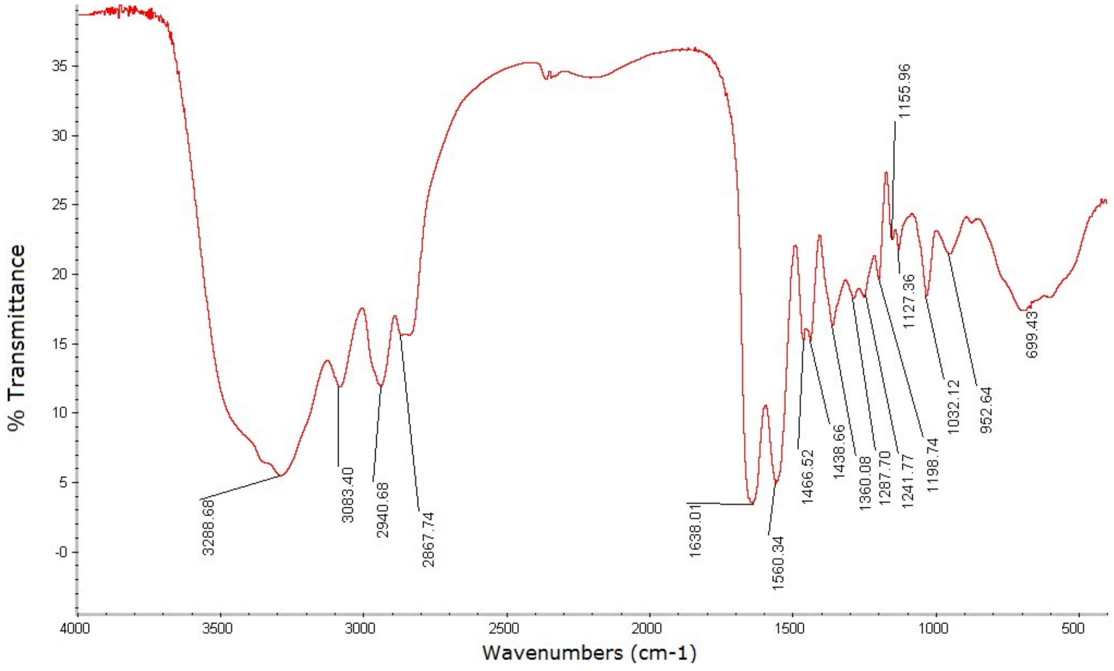

Fig. S4. FT-IR spectra of the synthesized hydroxyl-terminated G 5.0 PAMAM

dendrimer.

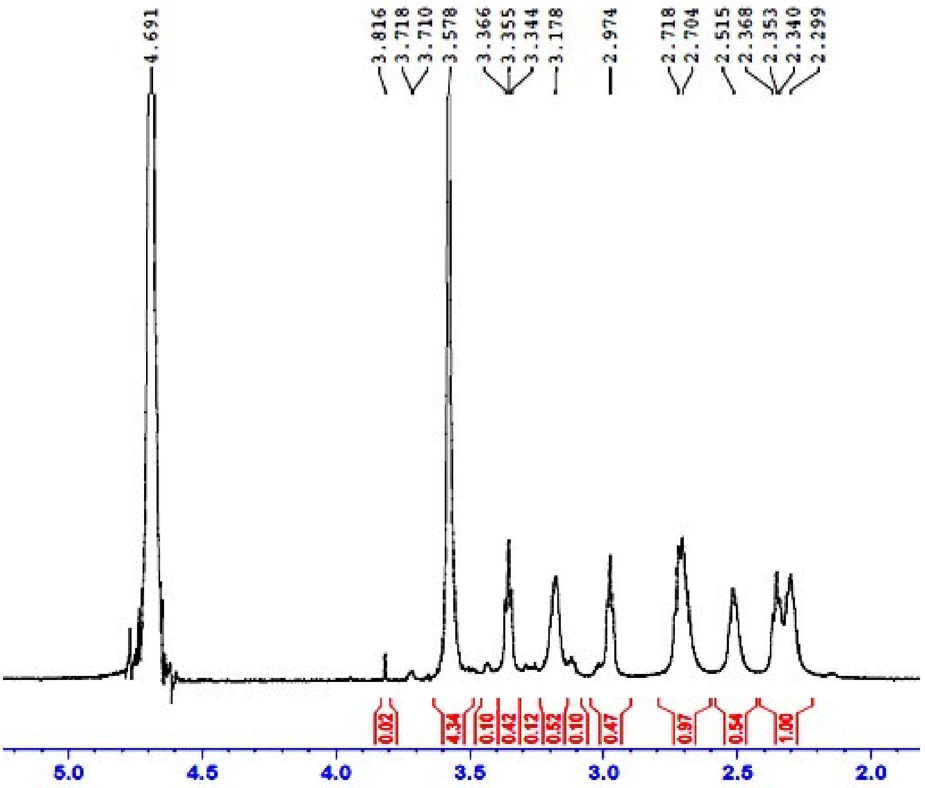

Fig. S5 ^{1}H-NMR spectra of synthesized PEGylated G5.0 PAMAM dendrimer

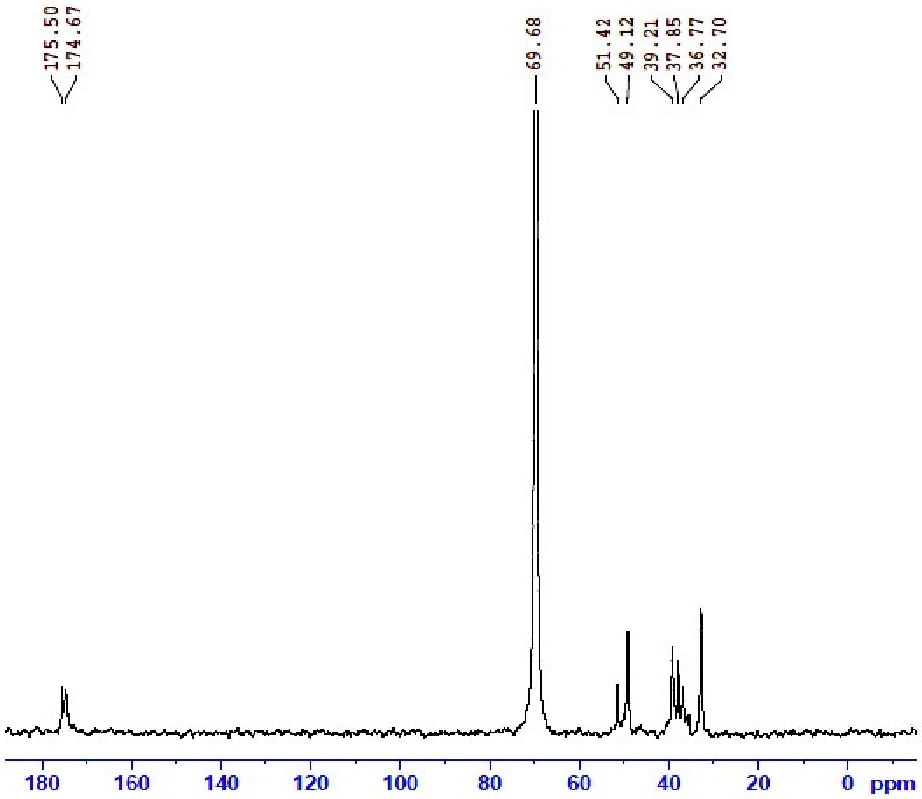

Fig. S6 ^{13}C NMR spectra of synthesized PEGylated G5.0 PAMAM dendrimer.

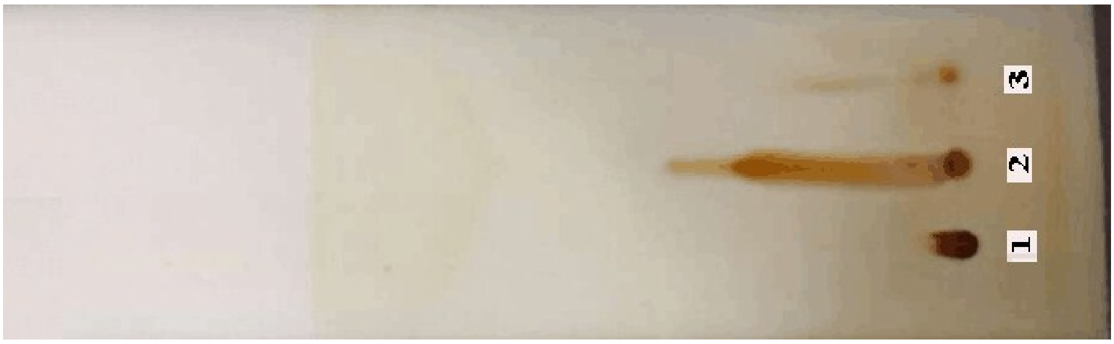

Fig. S7 Thin-Layer Chromatography (TLC) spectra of NHS-PEG-MAL-G5.0 PAMAM: chloroform-methanol (5: 1, v/v) was applied as TLC developing solvent for the separation of different components. The iodine steam was selected as chromogenic agent. Hydroxyl-terminated G5.0 PAMAM (lane 1), NHS-PEG-MAL-G5.0 PAMAM (lane 2) and MAL-PEG_{3400}-NHS (lane 3) were shown in this diagram, respectively.

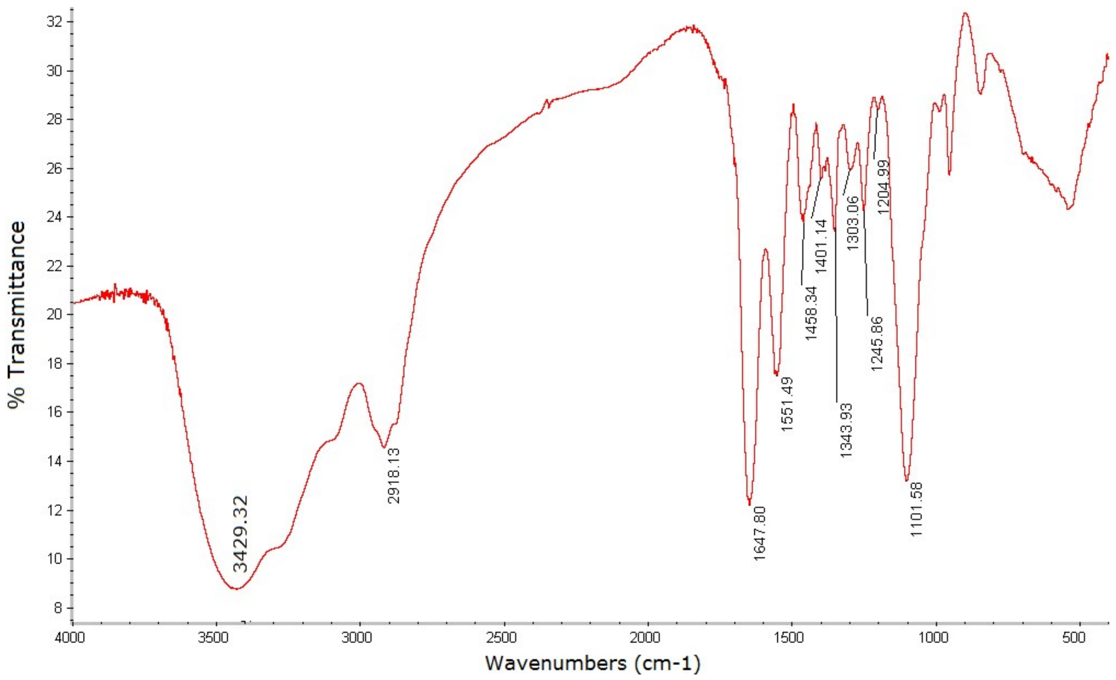

Fig. S8 FT-IR spectra of NHS-PEG-MAL-G5.0 PAMAM diblock copolymers

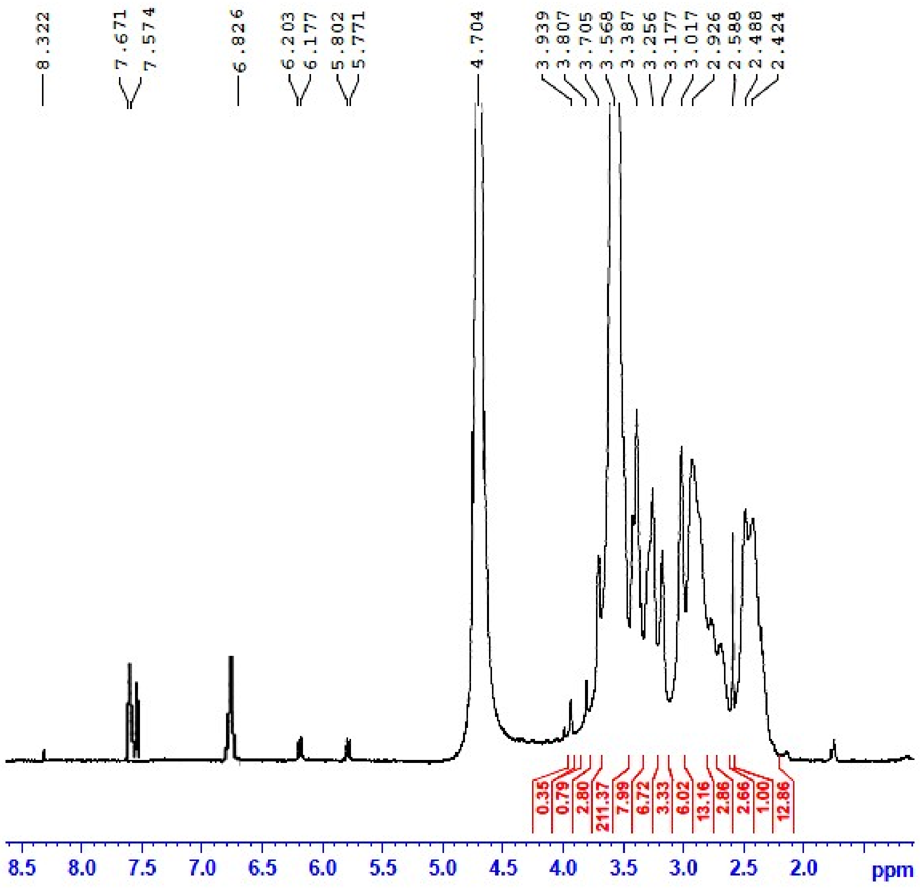

Fig. S9 ^{1}H NMR spectra of Angiopep-2-PGD-PEG-PAMAM

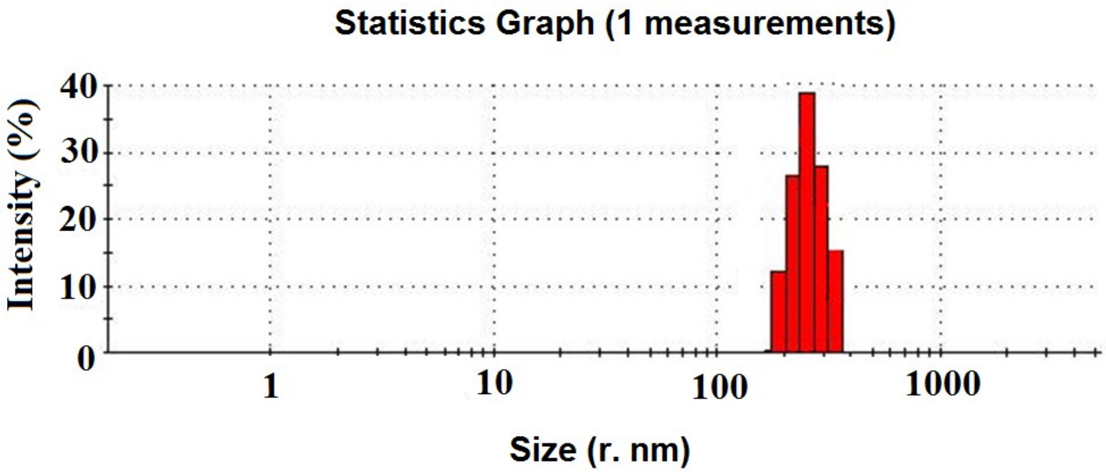

Fig. S10 Particle size distribution of Angiopep-2-PGD-STA-PEG-PAMAM NPs

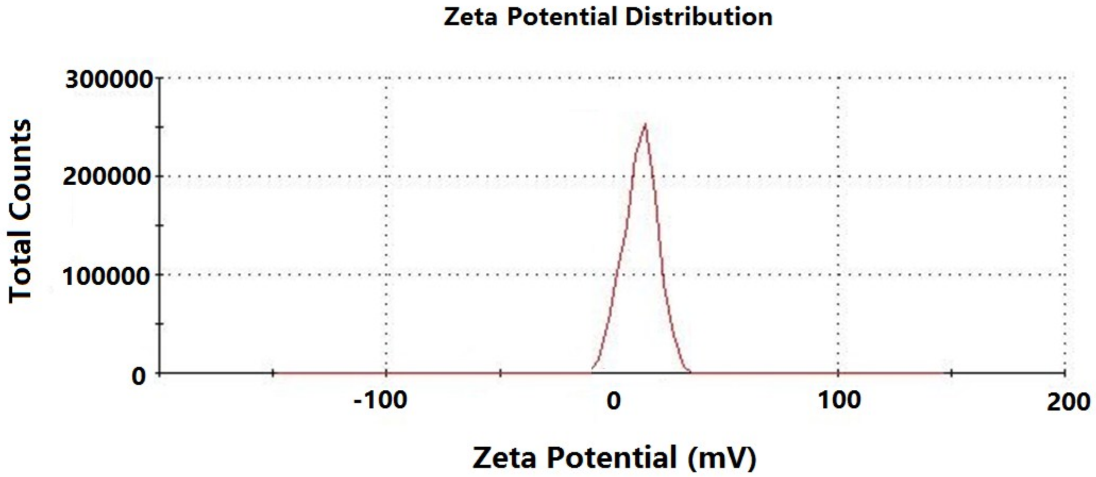

Fig. S11 Zeta potential distribution of Angiopep-2-PGD-STA-PEG-PAMAM NPs

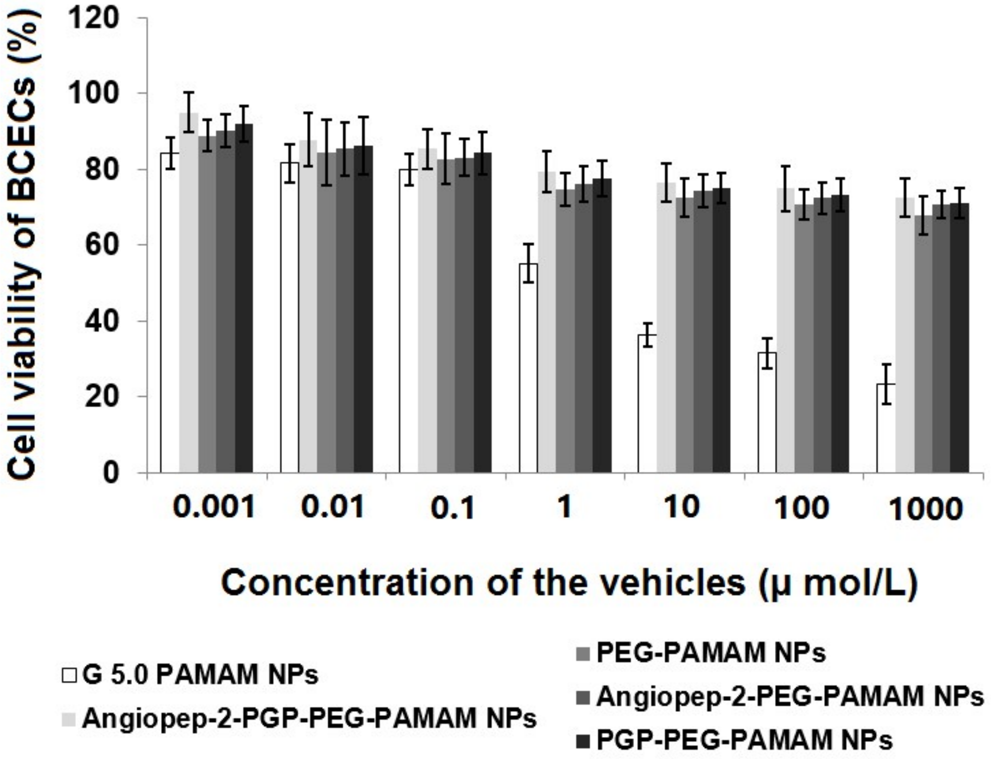

Fig. S 12 Cell viability of BCECs after treated with blank vehicles for 48 h at 37℃

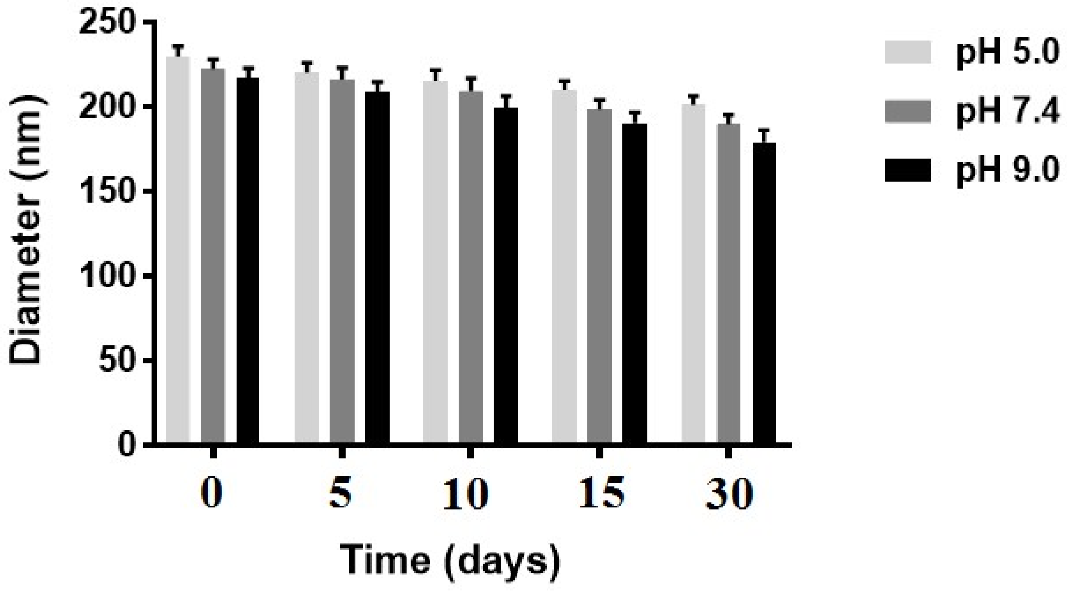

Fig. S13. The stability of Angiopep-2-PGP-PEG-PAMAM NPs in solutions with varying pH as judged by their diameter

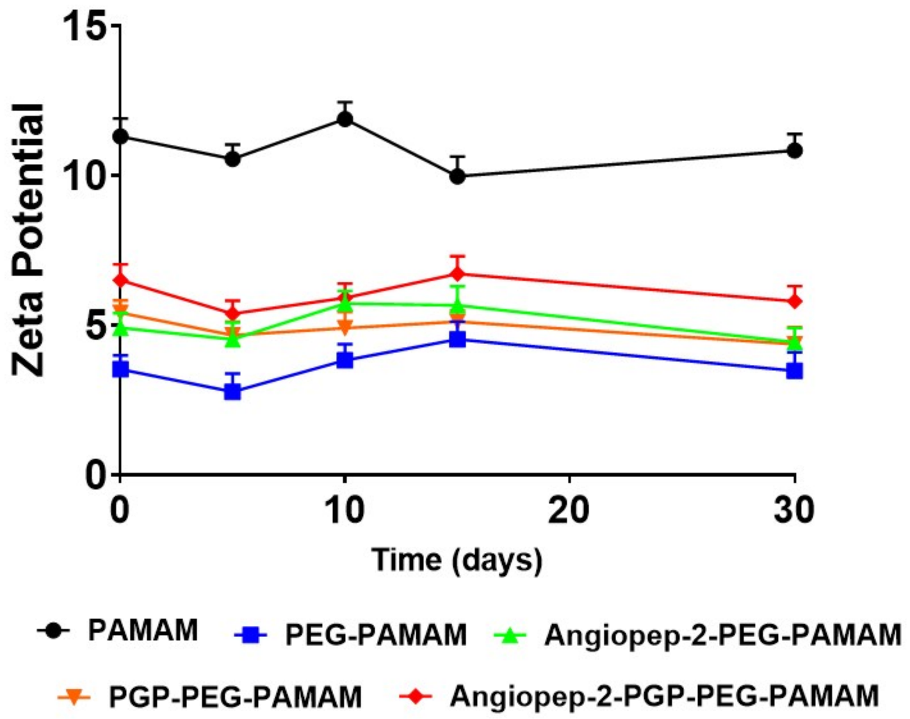

Fig. S 14 The stability of PAMAM, PEG-PAMAM, Angiopep-2-PEG-PAMAM, PGP-PEG-PAMAM and Angiopep-2-PGP-PEG-PAMAM in solutions as judged by their zeta potential

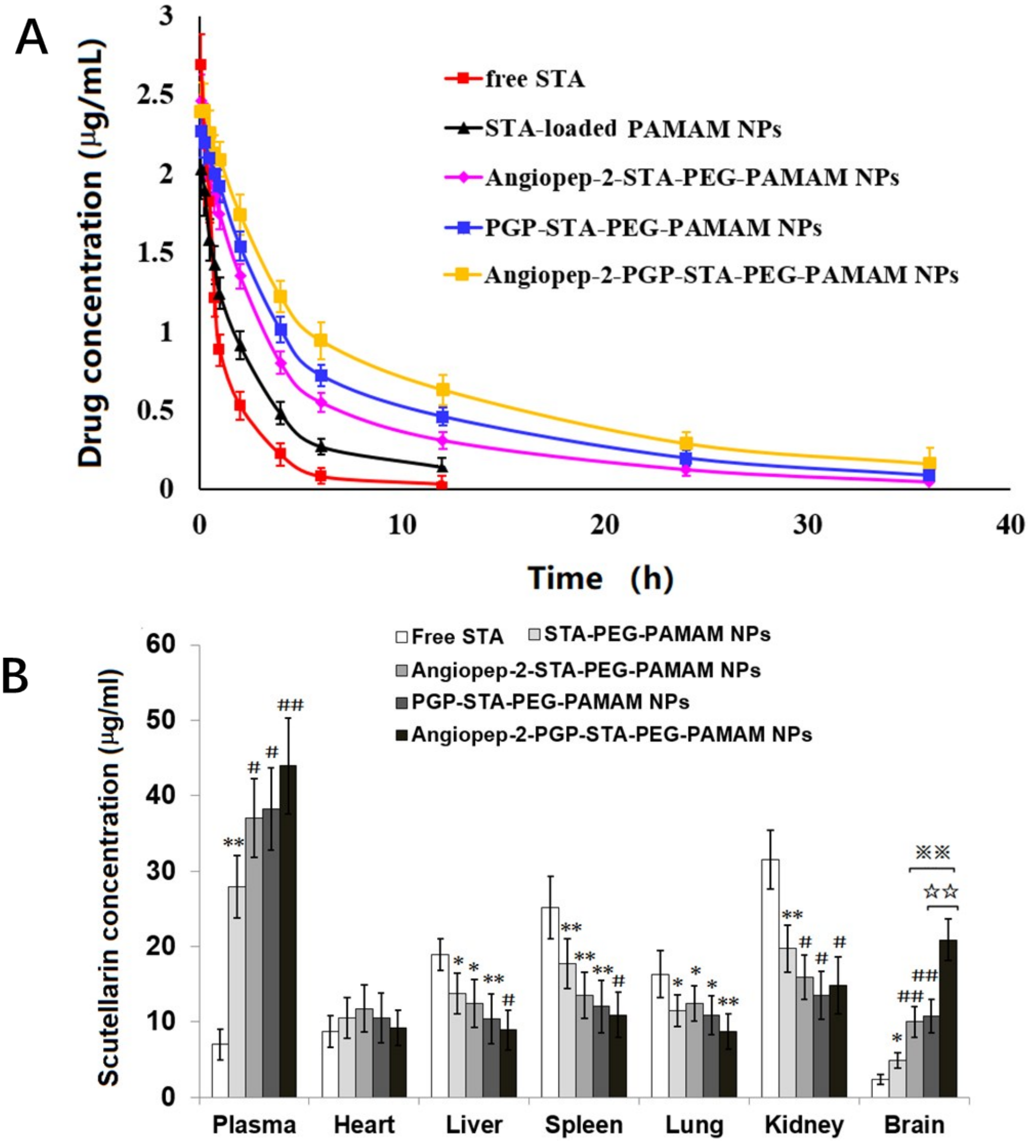

Fig. S15 Pharmacokinetics and biodistribution of dual-targeting nanoformulation in cerebral ischemia SD rats. (A) Plasma drug concentration-time profile of the dual-targeting STA-encapsulated nanoformulation after 36 h of intravenously injection compared with free STA, unPEGylated STA-encapsulated PAMAM NPs and single-ligand modified STA-encapsulated PEG-PAMAM NPs; (B) the bio-distribution of free STA, STA-encapsulated PEG-PAMAM-NPs, single-ligand modified STA-encapsulated PEG-PAMAM NPs and the dual-targeting STA-encapsulated nanoformulation after 12 h of intravenously injection. Statistically significant differences by Student's t-test when compared to the corresponding value of the control. Data represented mean±SD (n = 6).* $p<0.05$, ** $p<0.01$ vs. free

STA; ＃ p＜0.05, ＃＃p < 0.01 vs STA-encapsulated PEG-PAMAM NPs; ※※ p < 0.01 vs. Angiopep-2-STA-PEG-PAMAM NPs; ☆☆ p < 0.01 vs. PGP-STA-PEG-PAMAM NPs.

Table. S1 Sequences of the amplification primers used in the real-time PCR.

**mRNA Species**		**Oligonucleotides (5′→3′)**
HMGB1	forward	ATGGGCAAAGGAGATCCTA
	reverse	ATTCTCATCATCTCTTCT
NF-*κ*Bp65	forward	CGATCTGTTTCCCCTCATCT
	reverse	ATTGGGTGCGTCTTAGTGGT
IκB*α*	forward	TGGAGCCGACCTCAATAAACC
	reverse	TGCGACTGTGAACCACGATG
IKKβ	forward	GGAGTT TGGCATCACATCG
	reverse	GCCTCACCACCTCCT CTACT
TRAF6	forward	CGAAGAGGTCATGGATGCTAA
	reverse	TCAAAGCGGGTAGAGACTTCA
TLR2	forward	GAAAGATGCGCTTCCTGAAC
	reverse	CGCCTAAGAGCAGGATCAAC
TLR4	forward	GTTGGATGGAAAAGCCTTGA
	reverse	CCTGTGAGGTCGTTGAGGTT
TLR5	forward	CAGTTGCGAACCATAAGGACG
	reverse	GAGGTCACCGAGACAAAGCAC
MyD88	forward	ATTGGGGCAGTAGCAGATGAAG
	reverse	CAACCAGCAGAAACAGGAGTCT
IRAK-4	forward	CTGCTGCCAGATGCTGTTCC
	reverse	ACGGTTGTCCTGTTCTTTTCC
TRAM	forward	ATAAGTGCCCCCTTTCTTGG
	reverse	CCTCGTCGGTGTCATCTTCT
TRIF	forward	CAAGCCGTGCCCACCTACT
	reverse	TGTTCCGATGATGATTCCAG
GAPDH	forward	CAAGGTCATCCATGACAACTTTG
	reverse	GTCCACCACCCTGTTGCTGTAG

Table. S2 The main pharmacokinetic parameters of scutellarin (STA) following intravenous administration of free drug, Angiopep-2-STA-PEG-PAMAM NPs, PGP-STA-PEG-PAMAM NPs and Angiopep-2-PGP-STA-PEG-PAMAM NPs.

Pharmacokinetic parameters	free STA	STA -PAMAM NPs	Angiopep-2-STA -PEG-PAMAM NPs	PGP-STA-PEG-PAMAM NPs	Angiopep-2-PGP-STA-PEG-PAMAM NPs
$t_{1/2\beta}$ (h)	0.78 ± 0.05	0.91 ± 0.07	3.24 ± 0.44	3.51 ± 0.27	4.08 ± 0.56
MRT (min)	33.85 ± 3.57	45.73 ± 6.14	157.42 ± 11.54	162.38 ± 9.52	191.76 ± 10.62
AUC $_{0\sim\infty}$ (mg × h/l)	28.74 ± 2.73	37.39 ± 5.48	51.71 ± 9.34	53.41 ± 6.93	59.15 ± 8.76
CLs [L/(h kg)]	2.75 ± 0.18	2.41 ± 0.12	0.47 ± 0.06	0.46 ± 0.07	0.43 ± 0.04

References

1. I. J. Majoros, C. R. Williams, D. A. Tomalia and J. R. Baker, Jr., *Macromolecules*, 2008, **41**, 8372-8379.
2. A. Zarebkohan, F. Najafi, H. R. Moghimi, M. Hemmati, M. R. Deevband and B. Kazemi, *Eur J Pharm Sci*, 2015, **78**, 19-30.
3. Y. Jiang, P. Arounleut, S. Rheiner, Y. Bae, A. V. Kabanov, C. Milligan and D. S. Manickam, *J Control Release*, 2016, **231**, 38-49.
4. H. Guo, Q. Lai, W. Wang, Y. Wu, C. Zhang, Y. Liu and Z. Yuan, *Int J Pharm*, 2013, **451**, 1-11.
5. W. Zhang, M. W. Saif, G. E. Dutschman, X. Li, W. Lam, S. Bussom, Z. Jiang, M. Ye, E. Chu and Y. C. Cheng, *J Chromatogr A*, 2010, **1217**, 5785-5793.
6. X. Liu, C. An, P. Jin, X. Liu and L. Wang, *Biomaterials*, 2013, **34**, 817-830.
7. X. Liu, M. Ye, C. An, L. Pan and L. Ji, *Biomaterials*, 2013, **34**, 6893-6905.
